# Structural basis for control of integrative and conjugative element excision and transfer by the oligomeric winged helix–turn–helix protein RdfS

**DOI:** 10.1093/nar/gkaf249

**Published:** 2025-04-02

**Authors:** Callum J Verdonk, Mark Agostino, Karina Yui Eto, Drew A Hall, Charles S Bond, Joshua P Ramsay

**Affiliations:** School of Molecular Sciences, The University of Western Australia, Perth, WA 6009, Australia; Curtin Medical Research Institute, Curtin University, Perth, WA 6102, Australia; Centre for Crop and Disease Management, Curtin University, Perth, WA 6102, Australia; Curtin Medical Research Institute, Curtin University, Perth, WA 6102, Australia; School of Molecular Sciences, The University of Western Australia, Perth, WA 6009, Australia; Curtin Medical Research Institute, Curtin University, Perth, WA 6102, Australia; Curtin Medical Research Institute, Curtin University, Perth, WA 6102, Australia; Curtin Medical School, Curtin University, Perth, WA 6102, Australia; Australian National Phenome Centre, Murdoch University, Perth, WA 6150, Australia; School of Molecular Sciences, The University of Western Australia, Perth, WA 6009, Australia; Marshall Centre for Infectious Disease Research and Training, The University of Western Australia, Perth, WA 6009, Australia; Curtin Medical Research Institute, Curtin University, Perth, WA 6102, Australia; Curtin Medical School, Curtin University, Perth, WA 6102, Australia

## Abstract

Winged helix–turn–helix (wHTH) proteins are diverse DNA-binding proteins that often oligomerize on DNA and participate in DNA recombination and transcriptional regulation. wHTH recombination directionality factors (RDFs) associated with tyrosine recombinases, stimulate excision of prophage and integrative and conjugative elements (ICEs). RdfS is required for excision and conjugation of the *Mesorhizobium japonicum* R7A ICE, ICE*Ml*Sym^R7A^, which carries genes for nitrogen-fixing symbiosis. We show RdfS binds to DNA regions within the IntS attachment site (*attP*) and within the *rdfS* promoter, enabling RdfS to coordinate *rdfS*/*intS* expression and stimulate RdfS/IntS-mediated ICE*Ml*Sym^R7A^ excision. Several RdfS DNA-binding sites were identified. However, no consensus motif was apparent and no individual nucleotide substitutions in *attP* prevented RdfS binding. RdfS forms extensive helical filaments in crystals, with subunits contacting via a novel α1**-**helix absent in other wHTH-RDFs. RdfS oligomerized in solution in the absence of DNA. Molecular dynamics simulations supported a role for the α1**-**helix in oligomerization and compaction of nucleoprotein complexes. Removal of RdfS-α1 did not eliminate DNA-binding *in vitro* but reduced oligomerization and abolished RdfS-mediated ICE*Ml*Sym^R7A^ excision and conjugative transfer. We propose the novel RdfS-α1 mediated oligomerization enables RdfS to specifically recognize larger DNA regions with low primary sequence conservation through an indirect readout mechanism.

## Graphical Abstract

**Graphical Abstract:**
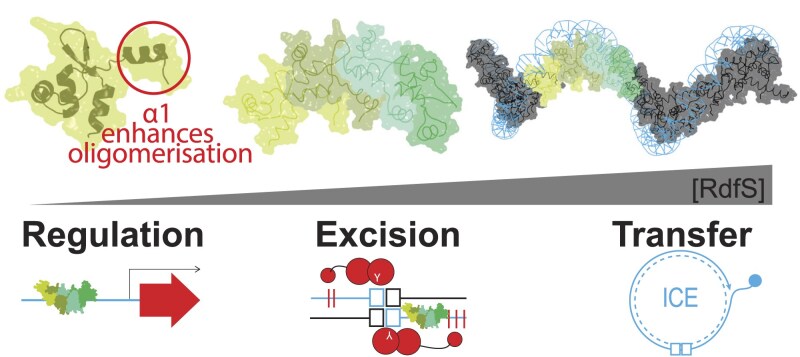

## Introduction

Integrative and conjugative elements (ICEs) are chromosomally integrating mobile genetic elements that often carry genes with selective advantages to their bacterial hosts, such as those necessary for biofilm formation, antibiotic and heavy metal resistances, virulence and pathogenicity, iron acquisition, and symbiotic nitrogen fixation [1–10]. Genome analyses indicate that ICEs are the most abundant type of conjugative element present in prokaryote genomes [11, 12]. ICEs reside integrated within the chromosomes of their hosts but can excise from the chromosome to form a transient circular element that is then able to transfer a single-stranded DNA copy through conjugation. Therefore, ICE excision and circularization is a prerequisite first step in ICE transfer. For most ICEs, it is unclear how the initiation of these distinct DNA recombination and conjugation processes are coordinated at a molecular level [13, 14].

Much like bacteriophage integration and excision, recombination between the ICE and bacterial chromosome is usually site-specific and catalyzed by a tyrosine or serine recombinase, also referred to as an integrase (Int). Integrases, together with specific DNA regions called attachment (*att*) sites, form nucleoprotein complexes that orchestrate DNA strand exchanges, facilitating both integrative and excisive recombination [15, 16]. Attachment sites for integrases of the tyrosine recombinase family contain a central core sequence that acts as the DNA substrate for strand cleavage and exchange. Recombination is catalyzed by the C-terminal recombinase domain of the integrase. During ICE integration, an attachment site *attP* present on the circular excised ICE is recombined with a corresponding chromosomal *attB* site. The *attP* region also contains specific ‘arm’ (also known as ‘P’) sequences flanking the recombined ‘core’ region. These arm sites interact with the N-terminal DNA-binding domain of the integrase and support the three-dimensional organization of the nucleoprotein complex during integration and excision reactions [17, 18]. Following ICE integration, recombined *attP* and *attB* sites produce chimeric *attL* and *attR* sites, each containing a copy of the core sequence and some of the arm sites derived from *attP*. The resulting recombined core sequences within *attL* and *attR* form direct DNA sequence repeats flanking the integrated ICE [15, 16, 19].

While integrase enzymes can catalyze both integrative and excisive recombination reactions, for most tyrosine recombinases, the formation of the integration products *attL* and *attR* is favoured. Excisive recombination often requires a recombination directionality factor (RDF) protein, also known as an excisionase. Tyrosine-recombinase RDFs are typically small positively charged DNA-binding proteins that bind DNA attachment sites and, in some cases, interact with integrase proteins directly [20–24]. While RDFs are extremely diverse at the primary amino-acid sequence level, they are frequently winged helix–turn–helix (wHTH)-domain proteins [20, 25]. wHTH proteins often also have roles as transcriptional regulators [26–33] and some are regarded to be nucleoid-associated proteins [34–38] that may perform roles analogous to eukaryotic histones; binding, bending, wrapping, and/or bridging along DNA to form large nucleoid complexes [39, 40].

The symbiosis ICE of *Mesorhizobium japonicum* R7A, ICE*Ml*Sym^R7A^, is a 502-kb ICE carrying genes necessary for nitrogen-fixation and symbiosis with *Lotus* spp. ICE*Ml*Sym^R7A^ encodes genes for root nodule formation, nitrogen fixation, metabolism, and protein secretion [6, 41, 42]. Integration and excision of ICE*Ml*Sym^R7A^ requires the tyrosine recombinase (integrase) IntS, which is encoded by the *intS* gene located immediately downstream of the *attL* site on the integrated ICE*Ml*Sym^R7A^ element. Experiments using a constructed ‘mini-ICE’ carrying only the *attP* region or the *attP-intS* region (on a plasmid unable to replicate in *Mesorhizobium*) reveal *attP* and *intS* are the only ICE*Ml*Sym^R7A^-encoded factors required for efficient integration. The 283-bp *attP* sequence contains five IntS P/arm-type sequence motifs (consensus TGKTGGTATC), and all five P/arm-type sites are required for efficient integration [43].

Most RDF genes are positioned nearby their cognate integrase genes. However, the ICE*Ml*Sym^R7A^ RDF gene *rdfS* is unique in that it is positioned as the first gene in an operon containing conjugation genes *traF*-*msi107* and is upstream of the origin of transfer (*oriT*) site and the conjugative relaxase gene *rlxS* [19]. This genetic organization of *rdfS* is conserved on numerous ICEs and plasmids found throughout the proteobacteria [14], suggesting the stimulation of ICE*Ml*Sym^R7A^ excision has become intertwined with the activation of conjugation. Interestingly, deletion of *rdfS* not only abolishes ICE*Ml*Sym^R7A^ excision, but also abolishes the ability of ICE*Ml*Sym^R7A^ to mobilize a plasmid carrying a cloned copy of the ICE*Ml*Sym^R7A^*oriT* [43], suggesting RdfS is also required for activation of conjugation.

Introduction of a plasmid overexpressing *rdfS* is lethal in *M. japonicum* R7A and only cells that have lost ICE*Ml*Sym^R7A^, named R7ANS, survive [19]. The R7ANS strain contains a repaired *attB* site resembling a naïve host capable of receiving ICE*Ml*Sym^R7A^. Introduction of the *rdfS* gene expressed on a plasmid stimulated complete excision and loss of the *attP*-*intS* mini-ICE from the cell population. Introduction of a similar plasmid over-expressing *intS* only moderately increases excision frequency (measured by *attP* abundance) and loss of the mini-ICE (*attB* abundance), suggesting both RdfS and IntS together are required to strongly stimulate IntS-mediated excision [44, 45].

Several ICEs related to ICE*Ml*Sym^R7A^ (referred to as the ICESym family [14]) exist in a tripartite configuration. These tripartite ICEs are present as three separate DNA sections in their host chromosomes. Tripartite ICEs assemble into a single circular element prior to conjugative transfer and then separate back into three parts integrated within the chromosomes of recipients [46]. The archetypical tripartite ICE, ICE*Mc*Sym^1271^ from *Mesorhizobium ciceri* WSM1271, encodes three site-specific tyrosine recombinases—IntS, IntG, and IntM. During ICE*Mc*Sym^1271^ integration, the circularized tripartite ICE, which carries three distinct *attP* sites, integrates into the chromosome at one of the three different *attB* sites and then recombines twice more with the chromosome to separate the ICE into three sections [46, 47]. Tripartite ICE excision is the reverse of this process and requires an orthologue of RdfS, RdfS_1271_, which shares 85% amino-acid identity with RdfS. ICE*Mc*Sym^1271^ encodes two additional RDF genes *rdfG* and *rdfM*, located adjacent to their cognate integrase genes *intG* and *intM*. Expression of RdfS_1271_ activates transcription from both the *rdfG* and *rdfM* promoters [44], thus coordinating all three excision reactions and formation of a single circular excised ICE*Mc*Sym^1271^ prior to transfer.

Other structurally characterized wHTH proteins have been shown to oligomerize in a head-to-tail fashion along DNA, forming variable-length nucleoprotein structures with pleotropic roles on transcription and recombination. In this work, we interrogate the previously solved RdfS crystal structure [45] to gain insight into the structural basis for the various functions of RdfS. RdfS oligomerized in solution even in the absence of DNA, and in crystals, RdfS oligomers have been shown to form an infinite superhelical polymer [45]. Compared to other wHTH proteins, RdfS carries an additional N-terminal α helix positioned at the core of the superhelical axis of RdfS polymers within crystals. DNA-binding assays confirmed that RdfS specifically bound DNA sites within *attP* and within the promoter regions of *rdfS* and *rdfG*. However, no obvious DNA-binding sequence motif was identified within these regions and no individual nucleotides within the *attP* site abolished binding. Removal of the unique N-terminal α-helix of RdfS diminished but was not essential for DNA binding *in vitro*; however, its removal abolished the ability of RdfS activate ICE*Ml*Sym^R7A^ excision and conjugative transfer. We propose that the unique N-terminal α-helix enhances RdfS oligomerization *in vivo* and that variable length RdfS oligomers specifically recognize diverse DNA sites on ICEs through an indirect readout DNA recognition mechanism.

## Materials and methods

### Microbiological techniques

Bacteria were cultured as previously described [19, 43, 48, 49] and further details can be found in the supplementary data. A list of strains and plasmids used in this study can be found in Supplementary Table S1. Oligonucleotides and synthetic DNA are listed in Supplementary Table S2. DNA extraction, plasmid construction, and β-galactosidase assays were carried out as previously described [49–52], and further information can be found in the supplementary data. ICE*Ml*Sym^R7A^ conjugation assays were carried out as described previously [46], with the exception that the R7ANS recipient strain carrying pPR3G was used in conjugation experiments and the selection medium contained gentamicin at 25 μg ml^−1^.

### DNA-binding assays

Electrophoretic mobility shift assays (EMSAs) were performed as described previously [49, 53]. DNA containing the ICE*Ml*Sym^R7A^*attP* and P*rdfS* were amplified by polymerase chain reaction (PCR) using IRDye800-labelled DNA oligonucleotides. Binding reactions were carried out using labelled DNA concentrations of 10 nM as described previously [49], and detailed in the supplementary data. Surface plasmon resonance (SPR)-based DNA-footprinting assays were performed using the Re-Useable DNA-Capture Technique (ReDCaT) [54] on the Biacore T200 (GE Healthcare) as described previously [49, 53] with protein concentrations at 1 μM. The oligonucleotide array for the *attP*, P*rdfS*, and *oriT* DNA regions were constructed using the Perl script *poop.pl* [54].

### Expression and protein purification

Purification of RdfS is described elsewhere [45]. 6HRdfS_1271_ and RdfS_13–89_ were purified using the same methods. All protein purifications were carried out on the ÄKTA pure chromatography system (GE Healthcare), using a 5 ml HisTrap column (Cytiva) for immobilized metal affinity chromatography and a HiLoad 16/600 Superdex 200 (GE Healthcare) for size-exclusion chromatography.

### Gel-filtration chromatography

Analytical gel-filtration was performed with a Superdex 75 10/300 GL (Cytiva) column equilibrated with size exclusion chromatography (SEC) buffer (50 mM Tris–HCl, 300 mM NaCl, 5% (*v/v*) glycerol; pH 7.4) on an ÄKTA pure chromatography system (GE Healthcare) at 4°C with a flow rate of 0.5 ml min^−1^. Each protein was concentrated to ∼7 mg ml^−1^ and injected in aliquots of 50 μl. Wavelengths of 230, 260, and 280 nm were used to visualize the interactions. Further details are available in the supplementary data.

### Small-angle X-ray scattering

Small-angle X-ray scattering (SAXS) data were collected on the SAXS/WAXS beamline at the Australian Synchrotron [55] with continuous data collection using a PILATUS 1M detector [56], using methods similar to those previously described [45]. Collection and processing parameters are shown in Supplementary Table S3. Further methodology can be found in the supplementary data.

### Molecular modelling and simulation

A model of RdfS binding to DNA was generated by adapting homology modelling methods previously described for generating DNA–protein complexes [57]. The model was generated through aligning monomers of RdfS (PDB 8DGL) to the complex of the *Streptomyces* protein BldC with DNA (PDB 6AMA) [36]. Molecular dynamics simulations were performed using GROMACS 2020.3 [58] patched with PLUMED 2.6.1 [59]. Full details of molecular modelling and simulations are described in the supplementary data.

### Quantitative PCR

Quantitative PCR assays to determine ICE*Ml*Sym^R7A^ excision rates were performed as previously described [19, 49]. Details of culture growth and primer efficiencies can be found in the supplementary data.

## Results

### RdfS is an oligomeric wHTH-domain with a unique N-terminal α-helix

**Figure 1.**
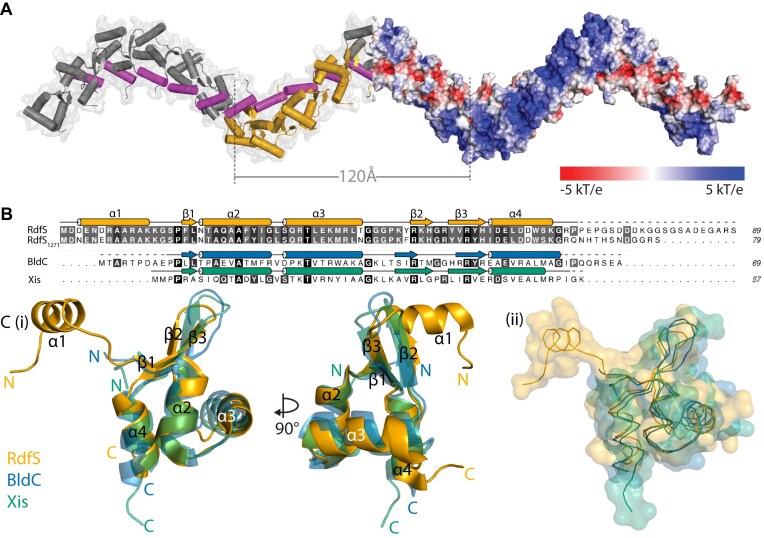
X-ray crystal structure of RdfS and comparisons to other wHTH-containing proteins. (**A**) Extended RdfS crystal structure revealing superhelical filaments. Electrostatic surface representation of 24 RdfS molecules is shown in red and blue at the right half of the helix, representing electronegative and electropositive regions, respectively (shown as a −5 to 5 kBT/ec gradient). The RdfS asymmetric unit of four molecules shown is depicted in cartoon format in yellow. The central α1 helix is shown in in magenta throughout the length of one full turn of the helix (eight RdfS molecules) spanning ∼120 Å. (**B**) Amino acid alignment of ICE*Ml*Sym^R7A^ RdfS and closely related *Mesorhizobium* RdfS homologue from *M. ciceri* WSM1271 (RdfS_1271_). Secondary structural representation shown above based on the X-ray crystal of R7A RdfS. Other HTH 17 grouped proteins included are *Streptomyces venezuelae* BldC [36] and Mycobacterium virus Pukovnik Xis [60] with their relative secondary structure features shown above each sequence. (**C**) (**i**) Ribbon model of RdfS (PDB 8DGL), BldC (PDB 6AMK), and Xis (PDB 4J2N) aligned to show similar features with each N- and C-terminus marked, and each location of the α-helices and β-strands for RdfS labelled to match panel *(B)*. (**ii**) Surface representation of RdfS, BldC, and Xis, with ribbon-backbone shown to highlight overall similarity between each structure [in same orientation as panel *(C) (i)*]. The RMSD between the wHTH domains of RdfS and BldC, and RdfS and Xis, are 1.66 Å (46 atoms) and 1.12 Å (39 atoms), respectively.

We recently solved the X-ray crystal structure for RdfS, revealing it forms a head-to-tail oligomer containing four RdfS molecules within the asymmetric unit (ASU), arranged with eight-fold helical symmetry within the crystal [45] (PDB entry 8DGL; Fig. 1A). The infinite head-to-tail oligomerization of RdfS protomers results in a superhelical protein filament extending throughout the crystal. Each of the four independent molecules observed share an effectively identical structure of four α-helices and a single three-stranded β-sheet: α1 (RdfS residues 3–12), β1 (17–18), α2 (19–28), α3 (30–40), β2 (47–49), β3 (52–56), and α4 (57–65) (Fig. 1B). In these crystals the C-terminal 21 residues (24%) of RdfS are completely disordered. The primary amino-acid sequence of RdfS (residues 17–66) is most similar to the group 17 HTH domain, which includes several other excisionase proteins (InterPro IPR041657). Alignments of the RdfS structure with Mycobacterium virus Pukovnik excisionase (RDF) Xis or the MerR-family *Streptomyces* BldC protein (Fig. 1C) exhibit Cα root mean square deviations (RMSD) of <2 Å.

**Figure 2.**
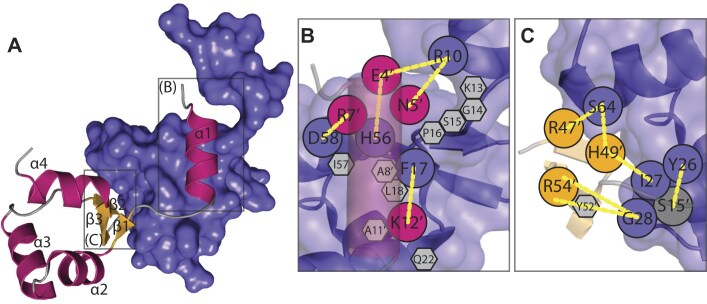
The interface between adjacent RdfS molecules in the crystal. (**A**) Ribbon diagram of RdfS molecule interacting with adjacent molecule as surface representation (purple). Ribbon colour scheme shown is strand; yellow, helix; magenta, and loop; grey. Schematic showing: (**B**) Close-up of RdfS N-terminus α-helix (α1) (transparent magenta cylinder) interacting residues. (**C**) RdfS β1–β2–β3 ‘wing’ region interacting with an adjacent RdfS protomer. Diagram residues coloured as in panel *(A)*, hydrogen bonds which stabilize the interface shown as yellow. Hydrophobic contact residues shown as grey hexagons.

The superhelical RdfS filament is stabilized by two protein–protein interfaces. The first interface involves the unique RdfS N-terminal α-helix (α1) from Asp3-Lys12, which sits in a hydrophobic pocket within the adjacent RdfS protomer (Fig. 2A). As far as the authors are aware, the α1 helix is absent from all other structurally characterized wHTH-domain proteins. The α1 charged sidechains from Glu4 and Arg7 form salt-bridges with the adjacent protomers’ charged side-chains His56 and Asp58, respectively. Hydrogen bonds are also formed between the α1 Asn5 and Lys13 sidechains interacting with the backbones of Phe17 and Arg10 from the neighbouring protomer. The α1-mediated interaction between molecules is primarily stabilized by the nine hydrophobic residues which span the surface of α1 and the neighbouring RdfS protomer (Fig. 2B). The overall surface area of α1 is 1286 Å^2^ and with an interface area of 463 Å^2^ with the adjacent RdfS molecule it is the major source of interaction area between RdfS molecules in the crystal structure. The second interface is between the wing region (β2–β3) of one RdfS molecule and the helices α2 and α4 of the neighbour; and with fewer hydrophobic residues at this interface, hydrogen bonding is the primary mediator of interface stability. The positively charged side-chains from strands β2 (Arg47 and His49) and β3 (Arg54) of RdfS interact with the backbone oxygens of α2 (Gly28 and Ile27) and α4 (Ser64) of the neighbouring molecule (Fig. 2C). This wing–α2/α4 interaction has a lower interface area of ∼400 Å^2^ compared with the α1’–RdfS interactions, suggesting that it may contribute more weakly to association.

Typically, wHTH proteins contact both the DNA major groove through their recognition helix and the DNA minor groove with their β-sheet ‘wing’. The positions of the expected major and minor DNA groove binding regions of the wHTH motif and electrostatic potential along the RdfS polymer are consistent with RdfS being capable of binding long stretches of double-stranded DNA (dsDNA) as an oligomer. The tentative recognition helix α3 contains exposed positively charged basic residues Arg32, Lys36, and Arg38 and exposed acidic residues Glu35 and Gln31, which may stabilize the protein–DNA interface. The arrangement of basic amino acids on the external surface results in a strongly positive electrostatic potential (Fig. 1A) and a continuous DNA-binding interface running the length of the superhelical protein filament. Superimposing RdfS monomers with a selection of crystal structures of wHTH:DNA complexes (Supplementary Fig. S1) revealed the orientation of the RdfS α3 and β2-β3 wing in the crystal structure resembles the orientation of DNA-binding components of other members of the wHTH family.

### RdfS binds the *attP* site upstream of *intS* and upstream of promoters P*rdfS* and P*rdfG*

**Figure 3.**
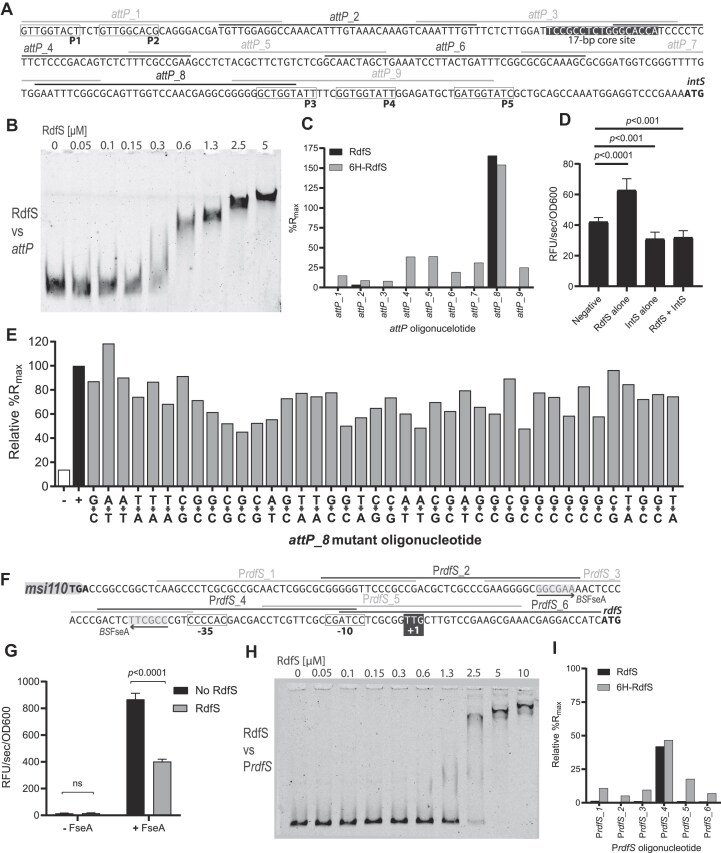
RdfS DNA-binding to the ICE*Ml*Sym^R7A^*attP* and P*rdfS*. (**A**) Sequence of the minimal ICE*Ml*Sym^R7A^*attP* DNA region required for integration, highlighting the arrangement of putative IntS-binding P sites P1–P5 (labelled boxes) and central recombination region (including the putative IntS ‘core’ site, white text, black background). Oligonucleotides (40 bp) used in SPR-based DNA-footprinting experiments are indicated by alternating grey and black lines above the *attP* sequence, labelled *attP_*1-*attP_*9. (**B**) EMSA using fluorescently labelled *attP* DNA (10 nM) and two-fold increasing concentrations of RdfS. (**C**) SPR DNA-footprinting for RdfS and 6H-RdfS using individual 40-bp oligonucleotides from the *attP* region. Oligonucleotide *attP*_8 consistently exhibited the largest %R_max_ response and was therefore used as a positive control in all subsequent SPR footprinting experiments. (**D**) β-Galactosidase activity in strains carrying a chromosomally integrated P*intS::lacZ* fusion (R7ANS::pFUS2P1-P5). Cultures were tested in the presence of RdfS (pSRKrdfS) and/or IntS (pJJ611), or with empty vectors (pSRKKm and pFAJ1708). (**E**) Analysis of SPR responses for dsDNA *attP_*8 oligonucleotides containing single base-pair substitutions. For each nucleotide position a substitution is indicated, where each base was switched for its complementary base on both strands of the dsDNA oligonucleotide. Each substitution was sequentially tested in SPR DNA-footprinting in a single run and the positive control *attP*_8 (+) and negative control *attP_*2 (−) oligonucleotides (shown as black and white bars) were run every 10th cycle and used to normalize drifting data over the duration of the experiment. (**F**) Sequence of the ICE*Ml*Sym^R7A^ P*rdfS* region. Each oligonucleotide tested in SPR DNA-footprinting are shown as alternating grey and black bars above the sequence (labelled P*rdfS*_1-P*rdfS*_6). The P*rdfS* transcriptional activator FseA binding site (*BS*FseA) [61] is shaded with a grey background and the inverted repeat are shown by arrows. The −35, −10 regions and +1 transcriptional start site for P*rdfS* estimated from previous 5′RACE experiments are indicated. (**G**) β-Galactosidase activity of stationary-phase R7ANS cultures containing P*rdfS-lacZ* (pSDrdfS-lacZ) plasmid constructs in the absence of FseA (‘− FseA’) or containing P*rdfS-lacZ* with the presence of FseA (pFseArdfS-lacZ: ‘+ FseA’). Cells additionally contained vectors pSRKKm (No RdfS: black) or pSRKrdfS (RdfS: grey). (**H**) EMSA with RdfS and the P*rdfS* region shown in panel *(F)*. (**I**) SPR DNA-footprinting of RdfS and 6H-RdfS against P*rdfS* 40-bp oligonucleotides. Values are shown as relative %R_max_ to the *attP*_8 control oligonucleotide (i.e. where *attP*_8 has a %R_max_ of 100%).

Characterized RDFs have been shown to bind specific DNA regions within their cognate *att* sites and promote excisive recombination catalyzed by their cognate integrase. Consistent with RdfS playing a similar role in ICE*Ml*Sym^R7A^ excision, overexpression of RdfS stimulates excision and loss of ICE*Ml*Sym^R7A^ from cells. Likewise, overexpression of RdfS in the presence of a mini-ICE plasmid carrying only the *attP*-*intS* region also results in loss of the mini-ICE, indicating that RdfS likely binds the *att* regions of ICE*Ml*Sym^R7A^ to stimulate IntS-mediated excision. To test whether RdfS could bind the *attP* region present on the circularized ICE*Ml*Sym^R7A^, RdfS was purified as an N-terminal hexahistidine fusion protein (6H-RdfS) and the 6H tag was removed following purification. EMSAs were carried out with the 283-bp *attP* region (Fig. 3A). EMSAs revealed RdfS retarded the migration of 10 nM *attP* when supplied at concentrations of 0.3–0.6 μM. The *attP* fragment was increasingly retarded with increased RdfS concentrations, suggesting that additional RdfS molecules were potentially oligomerizing on the RdfS–DNA complex (Fig. 3B). While the ratio of RdfS:DNA required to retard *attP* was relatively high, RdfS was unable to shift a 100-bp control DNA region (100-bp of the downstream *intS* gene) even with 10 μM of RdfS (Supplementary Fig. S2), confirming that RdfS binding to the *attP* region was specific. To refine the location of RdfS binding to *attP*, SPR-based DNA-footprinting assays were carried out. A tiled array of 40-bp double-stranded oligonucleotides spanning the *attP* region was designed, with each dsDNA oligonucleotide overlapping the sequence of the adjacent oligonucleotide by 10 bp (Fig. 3A). SPR assays with these oligonucleotides revealed that purified RdfS (1 μM) induced a strong binding response with a single dsDNA oligonucleotide, *attP*_8, the sequence of which overlaps the putative IntS arm site ‘P3’ towards the 3′ end of the *attP* region. The SPR binding response (%R_max_) values were 150%–250% of the theoretical R_max_ (across multiple experiments), suggesting more than one RdfS molecule was bound to *attP*_8. Due to the apparent complexity of RdfS–DNA interactions we were unable to determine association/dissociation kinetics from SPR data. Interestingly, when 6H-RdfS was used in SPR experiments, binding responses with *attP*_8 were unchanged; however, binding to each of the other oligonucleotides increased, suggesting that the 6H tag slightly increased non-specific DNA binding (Fig. 3C).

We attempted to further delineate the RdfS binding region within the *attP*_8 sequence by carrying out SPR experiments with a 20-bp oligonucleotide array covering the *attP_*8 sequence, with each oligonucleotide overlapping the adjacent oligonucleotides by 15 bp. However, none of these 20-bp dsDNA regions achieved >45% R_max_ observed for *attP*_8 (Supplementary Fig. S2), suggesting that either the optimal RdfS binding region in *attP*_8 was larger than 20-bp and/or that the positioning of the oligonucleotide did not capture the entire binding motif. To determine whether there were any critical nucleotides targeted by RdfS within the 40-bp *attP*_8 oligonucleotide, we created 40 new variants of *attP*_8 dsDNA oligonucleotide, each with a single-base-pair substitution to its complement nucleotide. Strikingly, no single nucleotide substitution abolished RdfS binding specificity, although some nucleotide changes reduced the relative %R_max_ to ∼50%–60% that of the native sequence (Fig. 3E). Together, these data suggest that RdfS oligomers may bind the *attP* sequence over a relatively large DNA-binding region, and that while the binding to this region is specific, no individual nucleotides in the region are absolutely critical for RdfS binding.

Due to the position of *attP*_8 sequence directly upstream of *intS*, we wondered whether RdfS and/or IntS might regulate transcription of the *intS* gene. In previous work, a plasmid pFUS2P1-P5 was constructed carrying the *attP* region with the *intS* gene replaced by *lacZ*, thus forming an *attL*-integrated transcriptional fusion carrying the P*intS* promoter [43]. Therefore, we used this strain to report on the expression of *intS* in the presence and absence of *rdfS*. Measured β-galactosidase expression from the P*intS*-*lacZ* fusion was increased ∼53% above background levels when *rdfS* was expressed from plasmid pSRKrdfS, indicating RdfS activates transcription of *intS* (Fig. 3D). In contrast, β-galactosidase expression was decreased ∼25% below background levels in the presence of the *intS*-expressing plasmid pJJ611, revealing that IntS negatively regulates its own expression. When both pSRKrdfS and pJJ611 were introduced, β-galactosidase expression was similar to the strain carrying the *intS-*plasmid pJJ611 alone, indicating that the negative autoregulation by IntS was dominant over the observed transcriptional activation by RdfS. In summary, the results of these experiments are consistent with a model where RdfS activates transcription from P*intS* and once IntS is produced, IntS negatively autoregulates its own expression.

Artificial overexpression of RdfS from a plasmid is lethal in R7A cells and leads to curing of ICE*Ml*Sym^R7A^ from cells [19]. However, we have never observed natural curing of ICE*Ml*Sym^R7A^ from cells during culture, suggesting that RdfS might negatively regulate its own transcription from P*rdfS* and prevent *rdfS* overexpression. To explore this, a previously constructed P*rdfS*-*lacZ* fusion was used to report on P*rdfS* transcription using β-galactosidase assays [50]. Since there is negligible expression from P*rdfS* in the absence of the transcriptional activator FseA, a plasmid containing both an IPTG-inducible copy of *fseA* and a P*rdfS*-*lacZ* transcriptional fusion was used to estimate transcription from P*rdfS* (Fig. 3G). A second plasmid carrying *rdfS* also under control of the *lac* promoter (pSRKrdfS) was used to express RdfS. As previously demonstrated, strong P*rdfS*-*lacZ* expression was observed from the pFseArdfS-lacZ plasmid because FseA activates transcription from P*rdfS*. However, when RdfS was additionally expressed from plasmid pSRKrdfS in the same cells, β-galactosidase activity was reduced ∼54%, indicating RdfS also binds P*rdfS* and attenuates the transcriptional activation by FseA.

**Figure 4.**
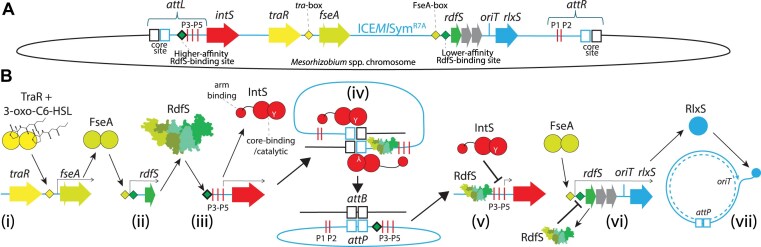
Stepwise induction of ICE*Ml*Sym^R7A^ excision and transfer coordinated by IntS and RdfS. (**A**) A simplified depiction of integrated ICE*Ml*Sym^R7A^ (light blue) including the *attL* and *attR* regions, the putative arm-type IntS-binding sites P1–P5 and other genes and binding sites involved in coordinating ICE*Ml*Sym^R7A^ excision (as labelled). (**B**) A stepwise model of ICE*Ml*Sym^R7A^ excision and transfer. (**i**) In transfer-active cells, the quorum sensing transcriptional activator TraR together with 3-oxo-C6-HSL binds a *tra*-box upstream of *fseA* and stimulates transcription (*fseA* is present as a two-part open-reading frame *msi172-msi171* and the FseA protein is translated following a programmed ribosomal frameshift). (**ii**) An FseA dimer binds the FseA box upstream of P*rdfS* and activates transcription of *rdfS* and downstream genes involved in transfer (*traF*, *msi107*, and *rlxS*). (**iii**) RdfS (shown as tetramer in shades of green), either as monomers or potentially as preformed oligomers bind the *attL* region of ICE*Ml*Sym^R7A^ upstream of the P3 arm-type IntS binding site. RdfS stimulates transcription of *intS* and production of IntS proteins. (**iv**) IntS proteins bind the core regions and arm-type sites present within *attL* and *attR* and together with RdfS, IntS catalyzes the excision of ICE*Ml*Sym^R7A^ through recombination of *attL* and *attR*, producing *attP* and *attB*. (**v**) Once excised, *attP*-bound IntS protein prevents further transcription of *intS*, likely keeping the ratio of IntS:RdfS low, preventing reintegration of ICE*Ml*Sym^R7A^. (**vi**) Once RdfS concentration increases above a certain threshold, RdfS binds the apparent lower-affinity binding site within P*rdfS*, repressing transcription from this promoter and thus preventing RdfS-mediated growth inhibition. (**vii**) Finally, following some unknown signal indicating a mating-pair has formed with a recipient cell, conjugation commences with RlxS nicking the *oriT* and piloting an ssDNA copy of ICE*Ml*Sym^R7A^ through the type-IV secretion system to the recipient cell.

EMSAs were performed with purified RdfS and a 162-bp region containing P*rdfS*. RdfS began to reduce migration of this DNA region when supplied at 1.3 μM. This concentration is approximately double the concentration of RdfS required to shift the *attP* region, indicating RdfS has an apparent weaker affinity for its own promoter than *attP* (Fig. 3H). We next again used SPR-footprinting with 40-bp tiled arrays of the P*rdfS* region to identify RdfS binding region(s). A single 40-bp dsDNA oligonucleotide P*rdfS*_4 was bound by RdfS. In comparison to the 40-bp *attP* region identified previously, the 40-bp *rdfS* binding response was 52% of the theoretical R_max_, which is ∼43% that of the binding response observed for the *attP*_8 oligonucleotide (Fig. 3I). The sequence of P*rdfS*_4 overlapped the −35 and −10 region of P*rdfS*, immediately downstream of the FseA binding site, consistent with RdfS repressing transcription by potentially occluding RNA polymerase from P*rdfS* (Fig. 3A). Shorter 20-bp dsDNA oligonucleotides derived from the P*rdfS*_4 sequence did not respond to RdfS in SPR experiments, again suggesting the optimal RdfS DNA binding site may be larger than 20 bp (Supplementary Fig. S2). Overall, the results of these experiments support a model of ICE*Ml*Sym^R7A^ excision in which RdfS first binds and stimulates *intS* transcription and stimulates IntS-mediated excision, after which IntS represses its own transcription from P*intS*. Once RdfS concentration increases above a certain threshold, RdfS binds P*rdfS* and represses its own expression, tentatively explaining why RdfS-dependent growth inhibition and loss of ICE*Ml*Sym^R7A^ has not been observed without artificial overexpression of *rdfS* (Fig. 4).

RdfS and RdfS_1271_ of the tripartite-ICE ICE*Mc*Sym^1271^ share 85% amino-acid identity over their aligned length and otherwise only differ in the length and sequence of their C termini (Fig. 1B). Previously we have demonstrated RdfS_1271_ activates transcription from the P*rdfG* and P*rdfM* promoters of ICE*Mc*Sym^1271^ [44], stimulated tripartite ICE excision through the sequential activities of integrases IntS_1271_, IntG, and IntM. To test if RdfS_1271_ exhibited similar DNA-binding characteristics to RdfS, hexahistidine-tagged RdfS_1271_ (6HRdfS_1271_) was expressed and purified from *Escherichia coli*. 6HRdfS_1271_ was tested in SPR experiments using the tiled oligonucleotide arrays designed for ICE*Ml*Sym^R7A^*attP* and P*rdfS* regions as shown in Fig. 3A and F. Similar to R7A RdfS, 6HRdfS_1271_ bound the ICE*Ml*Sym^R7A^*attP*_8 and P*rdfS*_4 oligonucleotides with the highest relative %R_max_ (Supplementary Fig. S3A), indicating an identical DNA-binding specificity on ICE*Ml*Sym^R7A^, despite RdfS_1271_ originating from ICE*Mc*Sym^1271^. Next, tiled oligonucleotide arrays were designed spanning the sequences of ICE*Mc*Sym^1271^ promoters P*rdfS*, P*rdfG*, and P*rdfM*. For the ICE*Mc*Sym^1271^ P*rdfS*, 6HRdfS_1271_ bound a dsDNA oligonucleotide in similar relative position within the P*rdfS*_1271_ promoter region as RdfS did with its cognate promoter (offset 5′ by 8 bp: Supplementary Fig. S3B) confirming both RdfS and RdfS_1271_ bind in similar positions overlapping their own gene promoters. 6HRdfS_1271_ bound two distinct regions within the P*rdfG* promoter region (Supplementary Fig. S3C). One of these was centred 170 bp upstream of the *rdfG* start codon, while the other was positioned directly upstream of the *rdfG* start codon, overlapping the putative ribosome-binding site. Only very weak binding was observed for any of the oligonucleotides in the P*rdfM* array (Supplementary Fig. S3D); however, this is consistent with very weak transcriptional activation of the P*rdfM* promoter by RdfS_1271_ compared to much stronger activation of the P*rdfG* promoter [44].

### RdfS oligomerizes alone in solution and together with DNA

**Figure 5.**
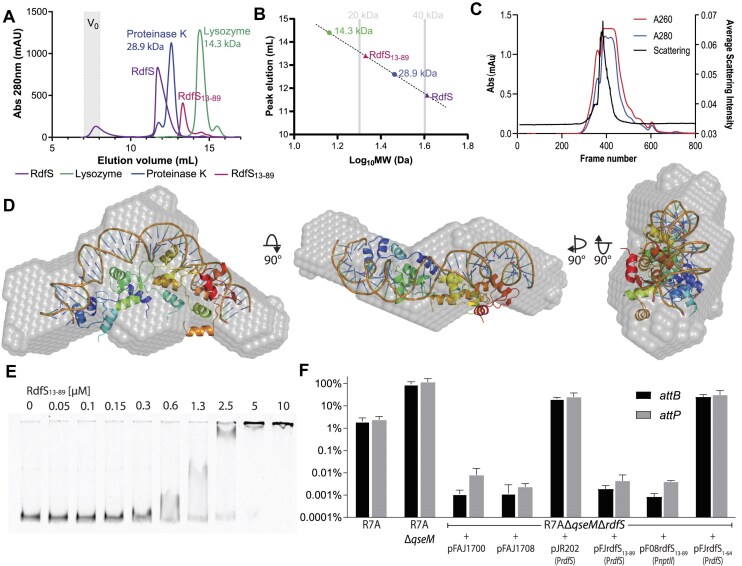
RdfS oligomerization, DNA binding and *in vivo* functions are impacted by deletion of α1. (**A**) Comparison of analytical size exclusion elution profiles of RdfS and RdfS_13–89_. RdfS elutes larger than 28.9 kDa, indicating likely oligomeric states of tetramers >40 kDa, with some soluble aggregation/large multimer complexes appearing at the void volume (V_0_; shown as grey). α1-truncated RdfS (RdfS_13–89_) elutes between 14.3 and 28.9 kDa in size, indicating dimers or trimers in solution (chromatography peak slightly larger than 20 kDa). (**B**) Log_10_ plot of SEC data from *(A)* for estimation of RdfS/RdfS_13–89_ complex sizes. (**C**) Ultraviolet elution profile showing absorbance at 260 (red) and 280 (blue) nm, and average intensity of scattering of the 6H-RdfS-*attP*_8 (black). Each frame represents 1 s of exposure. (**D**) Most representative (as determined by DAMAVER) DAMMIF *ab initio* envelope bead model of the 6H-RdfS-*attP*_8 complex, with a representative atomic structure of the RdfS–DNA complex from molecular dynamics simulations unbiased adiabatic model shown within. This atomized model excludes the extended N- and C-termini present in the scattering data and is only shown as a representative of a superficial visualization of a potential nucleoprotein arrangement fit to the *ab initio* envelope. (**E**) EMSA showing RdfS_13–89_ retarding the migration of the fluorescently labelled *attP* region. The N-terminal truncated RdfS_13–89_ remained capable of binding *attP* DNA with ∼50% the affinity of RdfS. Much like the EMSAs with RdfS, migration of *attP* was increasingly retarded with increasing concentrations of RdfS_13–89_, suggesting RdfS_13–89_ was still likely capable of oligomerizing on DNA with increasing concentrations. (**F**) The relative abundance (relative to the R7A chromosome copy number) of *attP* and *attB* (present only when ICE*Ml*Sym^R7A^ is excised) were measured by quantitative PCR to determine the frequency of ICE*Ml*Sym^R7A^ excision. Plasmids expressing RdfS or truncated RdfS variants RdfS_13–89_ and RdfS_1–64_ were introduced to determine if each variant was functional in activating ICE*Ml*Sym^R7A^ excision. The R7AΔ*qseM*Δ*rdfS* background was used as it is derepressed at a regulatory level for ICE*Ml*Sym^R7A^ excision and conjugation but lacks *rdfS* gene required to excise and transfer [52]. The promoter used to drive expression of each of the RdfS variants is shown in brackets under the name of each plasmid.

Most wHTH protein sthat form head-to-tail oligomers do so only in the presence of their cognate DNA sites [34]. The crystal structure of RdfS indicates that it can, at least in the crystal environment, form extensive oligomers in the absence of any DNA. We carried out analytical gel-filtration experiments to determine the oligomerization states of RdfS in solution. In the absence of DNA, RdfS eluted as an asymmetric peak with a leading edge corresponding to a maximum of ∼40 kDa (the expected mass of a tetramer) and a long trailing edge suggesting the presence of conformational or compositional heterogeneity (a flexible oligomer, and the presence of smaller oligomers such as trimers or dimers) (Fig. 5A and B). To validate the potential oligomeric properties of RdfS when binding DNA, we performed size-exclusion chromatography-coupled synchrotron SAXS (SEC-SY-SAXS) of 6H-RdfS bound to *attP*_8. Although there were multiple long trailing peaks for the 6H-RdfS-*attP*_8 sample in the SEC elution absorbance profiles, there was only one clear peak for the average X-ray scattering intensity profile (Fig. 5C), suggesting a single stable complex was being formed for 6H-RdfS-*attP_*8. Analysis of the 6H-RdfS-*attP_*8 complex indicated it shows properties of a monodisperse sample. The normalized Kratky plot indicates a flexible extended particle, with a peak above ∼1.1 and *q*R*_g_* beyond √3 returning towards zero at high *q*R*_g,_* indicating elongation [62–64], commensurate with the likely shape of a nucleoprotein complex (Supplementary Fig. S4). SAXS-derived molecular weight estimates using size and shape [65] indicated a 6H-RdfS-*attP*_8 mass of 85 655 Da, while concentration-independent estimation with Consensus Bayesian Assessment [66] suggested a complex mass of 83 125 Da (21.55% probable; credibility interval of 75 300–86 950 Da at 91.27% probability). These molecular weight estimates indicate a tetramer of 6H-RdfS bound to the dsDNA 40-mer *attP*_8; however, they predict a mass slightly larger than the expected molecular weight of 76.5 kDa, corresponding to four copies of 6H-RdfS and *attP*_8. The slight over-estimation for the molecular mass of the 6H-RdfS-*attP*_8 nucleoprotein complex is likely due to the increased scattering intensities of more electron-dense nucleic acids [67]. Generally adjusting for the difference in electron intensities for nucleic acids [68], the molecular weight estimates from the scattering approximates 71.5 kDa—within 7% of the expected molecular weights (Supplementary Table S3). *Ab initio* bead models from the scattering approximates an envelope shape which superficially matches a generated RdfS–DNA molecular dynamics model (Fig. 5D). While fits into these bead models are poor, this is unsurprising given the absence of the 6H tag and disordered C-termini in the molecular dynamics-generated RdfS–DNA model—which is reflected in the residuals at low and mid-*q* (Supplementary Fig. S4). We concluded from these data that RdfS is binding as a tetramer to the 40-bp *attP_*8 DNA target, suggesting 10-bp DNA per molecule of RdfS, comparable with previously characterized wHTH proteins which form multimers on DNA [36, 69–71].

### Molecular dynamics simulations suggest that the N-terminal helix stabilizes RdfS oligomers and compacts the RdfS quaternary structure

**Figure 6.**
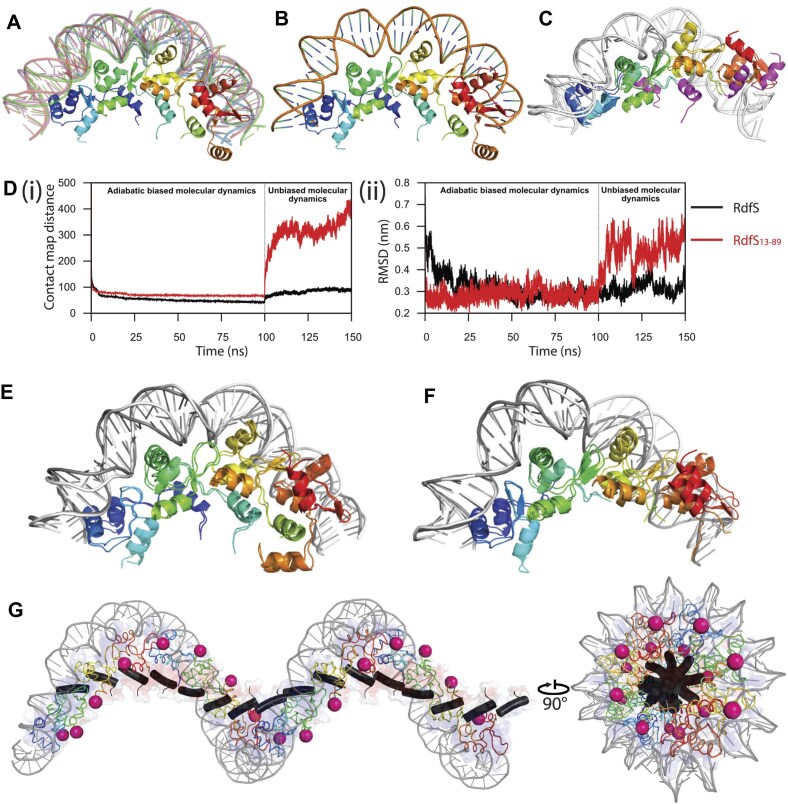
RdfS modelled with DNA. In all panels, the RdfS ASU tetramer is coloured in a blue-to-red rainbow from N- to C-terminal. (**A**) Visual representation of RdfS tetramer with an ensemble of fragments from crystallographically determined DNA for wHTH proteins: MtaN (red; PDB 1R8D), and filament forming wHTH proteins: BldC (blue; PDB 6AMK) and Xis (purple and green; 2IEF and 1RH6, respectively). Accompanying alignment to each structure individually can be found in Supplementary Fig. S1. (**B**) RdfS ASU tetramer modelled with nucleosome DNA (extracted from PDB 5GSE [72]) closely resembles the fragment ensemble model generated in panel *(A)*. (**C**) Overlayed models of DNA-bound RdfS and RdfS_13–89_ initially prepared via molecular modelling based on BldC-DNA (PDB 6AMA). The additional α1 (not present in RdfS_13–89_) shown in magenta. (**D**) (**i**) Distance in the contact map from the target state with respect to the RdfS crystal structure and (**ii**) RMSD over the adiabatic biased molecular dynamics (first 100 ns) and the subsequent unbiased simulation (final 50 ns) for DNA-bound full length RdfS (black) and RdfS_13–89_ (red). (**E**) Overlay of the representative structure of DNA-bound RdfS from adiabatic biased molecular dynamics (with DNA shown in white) with the representative structure following unbiased molecular dynamics simulation (with DNA shown in grey). See in-text and supplementary data for further molecular dynamics analysis. (**F**) Overlay of the representative structure of DNA-bound RdfS_13–89_ from adiabatic-biased molecular dynamics (ABMD) (with DNA shown in white) with the representative structure following unbiased molecular dynamics simulation (with DNA shown in grey). (**G**) Minimized model of extended RdfS filament (16 total molecules) coloured with electrostatic surface, and DNA derived from unbiased molecular dynamics simulation shown in panel *(E)*. RdfS α1 helix coloured in black and a magenta sphere marks the Cα of Pro66, showing the relative arrangement of the N- and C-termini of the RdfS superhelical structure.

Despite exhaustive attempts, we were unable to produce crystals of RdfS complexed with DNA, so we used *in silico* methods to assess how RdfS tetramers might bind with DNA targets and to probe whether the additional N-terminal α1 helix of RdfS might be important for these interactions. Extracting the DNA regions from wHTH-DNA nucleoprotein crystal structures (Supplementary Fig. S1) and superimposing them on the finalized RdfS ASU tetramer (Fig. 6A), we observed a strongly curved DNA bend path. To ensure this DNA bending was physically realistic, we first compared it to other structures with curved DNA and noted the bend to have similar parameters to nucleosomal DNA (Fig. 6B). We then performed molecular dynamics simulations on protein-only tetramers of both full length and N-terminally truncated RdfS_13–89_ (Supplementary Fig. S5A and B). The RMSDs (Supplementary Fig. S5C and D) over the simulation time courses for each tetramer suggest that RdfS_13–89_ yields a more flexible tetramer than the full length RdfS; this is further supported by cluster analysis, which indicates a vastly greater number of clusters for RdfS_13–89_ versus RdfS (Supplementary Table S4). The distance between the centres of mass of the capping molecules over the simulation time courses for each model, as well as within the middle structure of the largest cluster (Supplementary Fig. S5E and F), indicate that RdfS_13–89_ yields a slightly more compact tetramer compared to the full-length RdfS tetramer.

RdfS–DNA complexes for RdfS and RdfS_13–89_ generated by homology modelling (Fig. 6C) were subjected to adiabatic-biased molecular dynamics (ABMD), during which RdfS monomer-monomer interactions were targeted to replicate those observed in RdfS ASU tetramer (see supplementary data for full details). RdfS–DNA complexes showed structural similarity (RMSD ∼0.3 nm) to the protein-only RdfS crystal structure, indicating successful targeting of this protein assembly using ABMD (Fig. 6D). Subsequent unbiased simulations following the ABMD simulations suggested the general stability of full-length RdfS in this complex, maintaining a shape with similar curvature to nucleosomal DNA (Fig. 6E). In contrast, the complex with RdfS_13–89_ was conformationally unstable in unbiased simulations, with the distal two RdfS_13–89_ molecules dislodging from the DNA (Fig. 6F), suggesting that the α1 helix is required for stable protein–protein and/or protein–DNA interactions. Extension of this 40-bp DNA model to a continuous RdfS superhelix highlights the possibility that RdfS and DNA can form an extensive polymeric complex (Fig. 6G), where the N-termini assemble along the superhelical axis of the elongated nucleoprotein and the C-termini face outward toward the external surface, with an overall left-handed twist consistent with negative supercoiling.

### The N-terminal helix of RdfS is not essential for DNA-binding *in vitro* but is essential for *in vivo* activation of ICE*Ml*Sym^R7A^ excision and conjugative transfer

Noting the importance of contacts involving the N-terminal α1 helix from RdfS, and its influence on stable DNA binding revealed by both the crystal structure and the molecular dynamics simulations, we decided to test experimentally if α1 was essential for oligomerization or DNA binding. A construct expressing a truncation of RdfS with residues 2–12 removed (RdfS_13–89_) was generated and the resulting protein purified. Analytical gel filtration experiments revealed RdfS_13–89_ was present mostly as dimers with an approximate molecular mass at ∼20 kDa (Fig. 5A). EMSAs were then carried out with RdfS_13–89_ against the *attP* region. A concentration of 1.3 μM RdfS_13–89_ was required to shift *attP* DNA, which is approximately twice the concentration required for the wild-type RdfS to shift the same DNA. Interestingly, we observed no significant reduction of the multi-levelled migration effect (Fig. 5E), suggesting that additional RdfS_13–89_ molecules can oligomerize independently of α1 when binding DNA. Therefore, it seems that removal of the α1 helix reduces protein oligomerization, but DNA binding propensity is reduced, and further oligomerization likely still occurs in conjunction with DNA. This was not surprising given other wHTH domains do not have the unique RdfS α1 helix and yet are capable of oligomerizing on DNA, and RdfS maintains the same, albeit smaller, oligomerization interface as these other proteins.

As RdfS_13–89_ was still capable of binding DNA *in vitro*, we wondered if an allele encoding this truncated version would be capable of activating ICE*Ml*Sym^R7A^ excision and conjugative transfer. Versions of the *rdfS* gene encoding N- and C-terminal truncations in RdfS, each positioned downstream of the native *rdfS* ribosome-binding site and P*rdfS* promoter, were constructed and cloned into plasmid pFAJ1700. To test the ability of these truncated alleles to stimulate ICE*Ml*Sym^R7A^ excision, we introduced these plasmids into *M. japonicum* R7A and used quantitative PCR assays to measure ICE*Ml*Sym^R7A^ excision. The R7AΔ*qseM*Δ*rdfS* background was used as it is derepressed for activation of ICE*Ml*Sym^R7A^ excision and transfer through deletion of the antiactivator gene *qseM*, but incapable of ICE*Ml*Sym^R7A^ excision or transfer due to the deletion of *rdfS*. R7AΔ*qseM*Δ*rdfS* cells containing empty-vector controls exhibited population excision frequencies <0.01% (Fig. 5F). When R7AΔ*qseM*Δ*rdfS* was used as a donor in conjugation experiments, transfer of ICE*Ml*Sym^R7A^ was not detected (detection limit of 1.3 × 10^−9^) (Supplementary Table S5). In the presence of a plasmid carrying the wild-type RdfS allele expressed from P*rdfS* (pJR202), the proportion of chromosomes with an excised ICE*Ml*Sym^R7A^ increased to ∼20% and conjugative transfer of ICE*Ml*Sym^R7A^ was restored to an average of 1.2 × 10^−4^ exconjugants per donor. In contrast, no restoration of excision or conjugative transfer was observed in the presence of α1-truncated RdfS_13–89_ expressed from pFJrdfS_13–89_. We wondered if overexpression of this *rdfS*_13–89_ allele might be able to compensate for the reduced DNA-binding exhibited by this protein; however, even when *rdfS_13–89_* was expressed from the strong constitutive *nptII* promoter on pF08rdfS_13–89_, no restoration of excision or conjugative transfer of ICE*Ml*Sym^R7A^ was observed suggesting RdfS-mediated excision of ICE*Ml*Sym^R7A^ requires the RdfS α1-helix. We also constructed a separate plasmid coding for a version of RdfS deleted for the C-terminal 25 amino acids, RdfS_1–64_ (residues 64–89 are random coil or unresolved in the RdfS crystal structure). In contrast to the N-terminal truncation, C-terminally truncated version of RdfS restored excision and conjugation frequences to a similar level as full-length RdfS, suggesting that the disordered C-terminus of RdfS is not critical for excision and conjugation functions.

## Discussion

The diverse molecular roles of the RdfS homologues encoded by *Mesorhizobium* spp. ICEs have been previously reported [19, 43, 44]; however, their structural basis has remained uncharacterized. In this work, we analysed the X-ray crystal structure of *apo* RdfS and discover that the left-handed superhelical filaments formed in crystals are also represented in solution where, at the concentrations tested, RdfS forms tetramers both in the presence and absence of DNA. The head-to-tail oligomerization of RdfS molecules is likely enhanced by the unique N-terminal α-helix not present in other characterized wHTH proteins. DNA-binding assays confirmed RdfS bound the ICE*Ml*Sym^R7A^*attP* site within a 40-bp region directly upstream of the putative IntS P3 arm-site, supporting its role as an RDF/excisionase for IntS-mediated ICE*Ml*Sym^R7A^ excision. RdfS binding to *attP* also activated transcription from the P*intS* promoter, which is likely silenced immediately following ICE*Ml*Sym^R7A^ excision through negative autoregulation by the IntS protein. DNA binding assays revealed RdfS also binds its own promoter P*rdfS* and thus negatively autoregulates its own expression. The apparent weaker affinity of RdfS for P*rdfS* likely facilitates the establishment of an equilibrium concentration of RdfS in transfer-active cells, which ensures ICE*Ml*Sym^R7A^ excision and conjugation functions are activated without causing the growth inhibition and ICE*Ml*Sym^R7A^-curing observed following artificial *rdfS* overexpression. Molecular dynamics simulations predicted the unique N-terminal α1-helix of RdfS was likely required for optimal oligomerization and compaction of higher-order RdfS–DNA complexes. Interestingly, removal of the α1-helix reduced DNA binding and oligomerization *in vitro*, but this same modification abolished all detectable activity of RdfS *in vivo*. In summary, the RdfS protein is a unique member of the wHTH family with a novel oligomer-stabilizing α1-helix. We propose that RdfS oligomerizes in a concentration-dependent manner and such oligomerization facilitates the stepwise binding to various target sites required for ICE*Ml*Sym^R7A^ excision and conjugation functions.

The identified RdfS target region *attP_*8, overlaps the presumed promoter region of *intS*. Our P*intS-lacZ* fusion experiments revealed RdfS activates *intS* transcription. In isolation it could be interpreted that the sole role of RdfS therefore is to transcriptionally activate IntS expression rather than participate as RDF. However, overexpression of IntS alone does not stimulate the observed ∼100% ICE*Ml*Sym^R7A^ excision when both RdfS and IntS are expressed together [19], confirming RdfS is required to stimulate excision directly as observed for other RDFs [15, 20, 73, 74]. Our experiments also showed IntS negatively regulates its own expression. This presumably prevents RdfS from overinducing P*intS*, which could shift the ratio of RdfS:IntS in favour of ICE*Ml*Sym^R7A^ integration. Therefore, our data are consistent with a model in which RdfS expression transiently activates IntS expression and then IntS and RdfS together excise ICE*Ml*Sym^R7A^, after which IntS expression is inhibited by IntS, which a ratio of RdfS:IntS that maintains ICE*Ml*Sym^R7A^ in an excised state in preparation for conjugative transfer.

Other RDFs such P2 Cox [75], phage 186 Apl [76], P4 Vis [77], WΦ Cox [70], and P22 Cox [78] form higher-order complexes on *att* DNA. The increasing DNA retardation with RdfS concentration observed in EMSAs here supports RdfS forming variable-length multimers along the *attP* DNA region. Our SPR experiments revealed RdfS exhibits specificity for a 40-bp region *attP*_8 and smaller portions of this DNA region were not bound as strongly, suggesting the natural footprint for RdfS binding may be close to this size (at least at this site). SEC-SY-SAXS analysis of 6H-RdfS indicated a stable complex with *attP*_8 as a tetramer (4 RdfS: 1 *attP*_8 dsDNA), with one RdfS molecule per 10-bp of DNA. Preparations of purified RdfS tended to precipitate at higher concentrations, suggesting larger oligomers may form in solution but are unstable in the conditions tested. It seems possible that in more suitable conditions RdfS may form larger multimers together with longer DNA regions.

Much like RdfS, the P2 bacteriophage RDF Cox_P2_ is a wHTH domain protein that oligomerizes *in vitro* and its oligomerization is essential for its roles in P2 recombination and regulation [75]. However, Cox_P2_ encodes an additional C-terminal helix beyond the corresponding α4 helix in RdfS (Supplementary Fig. S6). The two C-terminal α-helices of Cox_P2_ enable it to form a continuous left-handed superhelical structure in crystals. This arrangement of Cox_P2_ enables the formation of oligomeric filaments which compact on DNA, mimicking histone architecture [79]. It seems likely that the C-terminal helices of Cox_P2_ and the N-terminal α1 helix of RdfS function in an analogous manner, enabling protein–protein contacts along DNA to compact and transform overall morphology of the nucleoprotein filament. Much like the removal of the α1 helix of RdfS, mutations within the C-terminal helices of Cox_P2_ abolish biological activity [24]. It seems possible that like the Cox_P2_ C-terminal helices, the RdfS α1 helix may facilitate nucleoprotein contraction and that this contraction may be critical to its functions as an RDF and a transcription factor. Such DNA compaction is supported by our molecular dynamic simulations (Fig. 6), although remains experimentally untested.

Despite identifying several DNA-binding targets for RdfS, we were unable to identify consensus binding motifs. Our SPR experiments also indicated no individual nucleotide changes abolished RdfS binding to *attP_*8, although most changes reduced the binding response. The *Streptomyces* BldC protein is a wHTH-domain regulator with structural similarity to ICE*Ml*Sym^R7A^ RdfS (Fig. 1). BldC binds multiple DNA targets and oligomerizes in a head-to-tail manner. Whilst BldC can bind a 22-bp DNA-target with 9-bp direct repeats (*whiI*) as a dimer, the mechanism of binding to the larger pseudo-continuous *smeA-sffA* promoter differs. BldC cooperatively forms extended filaments along the *smeA-sffA* region with one DNA-turn between each bound BldC molecule [36]. This resembles our proposed model of DNA binding by RdfS (Supplementary Fig. S6). Interestingly, binding of BldC to the *smeA-sffA* region appears not to be strictly dependent on DNA sequence-consensus, suggesting the target DNA structure could be more crucial than sequence—a phenomenon referred to as indirect readout [80, 81]. A similar indirect readout type of DNA-binding mechanism is described for another BldC orthologue from *Actinoplanes missouriensis*, which binds AT-rich regions [82], and for phage λ Xis which forms cooperative filaments along DNA [69]. Unlike RdfS however, *Streptomyces* BldC remains a monomer in solution and the multimeric superhelical behaviour is dependent on the presence of DNA—indicating BldC must be anchored to its cognate DNA to assemble into higher-order oligomers [36]. BldC primarily forms its head-to-tail oligomers due to its hydrophobic core and cross-contacts between Glu and Arg between protomers at the β wing-HTH region. Although the relative position of these residues differs in RdfS, equivalent contacts are made at the β wing-HTH, although many more RdfS intermolecular contacts are made with the α1 (Fig. 2B and C). Our analytical SEC and molecular dynamics suggest that RdfS oligomerization is mediated independent of DNA and RdfS–DNA binding stability is enhanced by the N-terminal α1 helix. Our data here indicate that RdfS can oligomerize in solution, so it seems possible that RdfS may oligomerize *in situ* prior to DNA binding and as such, have the capability to recognize larger and more dispersed DNA recognition motifs.

Evolving a capacity to recognize larger DNA recognition sites would presumably afford RdfS with a larger DNA footprint within which binding specificity could evolve. Such a capability may have been critical for the evolutionary success of ICE*Ml*Sym^R7A^ in competition with the multitude of related ICEs present in the genus *Mesorhizobium*. Proteins closely related to RdfS are encoded by conjugative elements throughout the genus *Mesorhizobium*. Indeed, all identified ICEs (average 1.2 ICEs per genome [14]) and most identified conjugative plasmids in *Mesorhizobium* carry a homologue of RdfS encoded in a similar genetic context upstream of the transfer genes *traF* and *rlxS*. Phylogenetic comparisons of the core conjugation/transfer genes of all *Mesorhizobium* ICEs places all symbiosis-gene carrying ICEs (both monopartite and tripartite) into a monophyletic group [14], which we have named the ICESym family. *Mesorhizobium* ICEs outside this clade exhibit a more diverse evolutionary history but nonetheless carry the same set of core conjugation/transfer genes, including *rdfS*. Strikingly, we observed here that only RdfS homologues encoded by the ICESym family contain the α1 helix (Supplementary Fig. S7). All other RdfS homologues encoded by *Mesorhizobium* ICEs and plasmids, while exhibiting ∼61% similarity over the wHTH domain region with ICE*Ml*Sym^R7A^ RdfS, do not encode the α1 helix. We speculate therefore that evolution of this helix coincided with the expansion and dissemination of the ICESym family throughout the genus *Mesorhizobium* and that the additional RdfS helix may have reduced regulatory interference from other RdfS homologues present in *Mesorhizobium* genomes. This model is also consistent with the broader evolutionary distribution of ICESym family ICEs compared to other ICE families in the genus [14, 83].

Several lines of evidence point to RdfS having a role in the regulation of conjugation. The position of the *rdfS* gene upstream of *traF* and *rlxS* conjugation genes is conserved across related ICEs and plasmids throughout the proteobacteria [14, 19]. This contrasts with nearly all other identified RDF genes, which are typically located adjacent to their cognate integrase gene [20]. Deletion of *rdfS* also abolishes ICE*Ml*Sym^R7A^ conjugation; however, this is not surprising given the requirement for ICE*Ml*Sym^R7A^ excision. More surprising is that the *rdfS* deletion also abolishes the ability of the ICE*Ml*Sym^R7A^ conjugation system to mobilize a plasmid carrying the ICE*Ml*Sym^R7A^*oriT*, a process which does not physically require ICE*Ml*Sym^R7A^ to excise. This suggests RdfS more directly activates conjugation functions. Furthermore, in these experiments, low-level expression of *rdfS* from the *lac* promoter restored ICE*Ml*Sym^R7A^ excision but did not restore conjugation [43], suggesting conjugation might require a greater level of *rdfS* expression. In conjugation experiments here, we introduced *rdfS* expressed from its native promoter and in contrast to our previous work, this restored both ICE*Ml*Sym^R7A^ excision and conjugation. These observations suggest that a greater concentration of RdfS is required to activate conjugation than stimulate excision. We speculate that the RdfS regulon extends beyond the *attP* and P*rdfS*/P*rdfG* regions identified in this work and that at higher concentrations RdfS activates or derepresses additional ICE genes more directly involved in conjugative transfer. The concentration-dependent binding and oligomerization of RdfS with various DNA targets described in this work fits well with such a model. In future experiments, genome-wide protein–DNA interaction assays such as ChIP-Seq in conditions of transient RdfS overexpression may unveil additional targets of the multifunctional RdfS.

## Supplementary Material

gkaf249_Supplemental_File

## Data Availability

Small-angle x-ray scattering data and fits were deposited at SASBDB under the accession code SASDW97 (https://www.sasbdb.org/data/SASDW97/).
