# Supplementary material for: Structural basis for control of integrative and conjugative element excision and transfer by the oligomeric winged helix–turn–helix protein RdfS
Source: Nucleic Acids Res. 2025 Apr 2;53(6):gkaf249. doi: 10.1093/nar/gkaf249 (PMC11963761; doi:10.1093/nar/gkaf249)
Supplement: gkaf249_Supplemental_File [file gkaf249_supplemental_file.pdf]

Supporting Information

Verdonk, CJ, *et al.* **Structural Basis for Control of Integrative and Conjugative Element Excision and Transfer by the Oligomeric Winged-Helix-Turn-Helix Protein RdfS**

Supplemental methods **2**
Media 2
β-galactosidase assays 2
Electrophoretic mobility shift assays 2
Small angle X-ray scattering 3
Quantitative PCR 3
Molecular modelling and simulation 4
Supplemental Figure S1 **7**
Supplemental Figure S2 **8**
Supplemental Figure S3 **9**
Supplemental Figure S4 **10**
Supplemental Figure S5 **12**
Supplemental Figure S6 **13**
Supplemental Figure S7 **14**
Supplemental Table S1 **15**
Supplemental Table S2 **17**
Supplemental Table S3 **23**
Supplemental Table S4 **25**
Supplemental Table S5 **26**
Supporting information references **27**

Supplemental methods

Media

Escherichia coli cultures were grown at 37°C in lysogeny broth (LB). *Mesorhizobium japonicum* strains were cultured at 28°C in tryptone yeast (TY) medium, buffered TY medium (TY with 40 mM MES pH 6.5), or glucose rhizobium defined medium (G/RDM) as previously described (1-3). Media were supplemented with antibiotics at the following concentrations: kanamycin (Km) 50 µg mL^{1}, neomycin (Nm) 200 µg mL^{-1}, gentamicin (Gm) 50 µg mL^{-1} (*E. coli*) and 25 µg mL^{-1} (*M. japonicum*), tetracycline (Tc) 10 µg mL^{-1} (*E. coli*) and 2 µg mL^{-1} (*M. japonicum*). Isopropyl β-D-1-thiogalactopyranoside (IPTG) was used at a concentration of 100 µM for protein expression and 200 µM for *Mesorhizobium* cultures carrying pSDz constructs.

β-galactosidase assays

Buffered TY liquid cultures were grown to stationary phase and sterile glycerol was added to a final concentration of 15% *v/v*. Aliquots were frozen and stored at -80°C overnight for use as seeder cultures. TY cultures were then inoculated with 1 in 200 dilution of seeder culture, IPTG was supplemented to a final concentration of 0.2 mM if applicable and grown to late stationary phase (~64 hours) with shaking. β-galactosidase assays were performed as described previously (4,5). For freeze-thawing, the plate containing cultures was frozen at -80°C (10 minutes) and thawed at 37°C (lid removed, 10 minutes), repeated three times. Each assay contained 10 µL culture in a final volume of 100 µL. An EnSight Multimode Plate Reader (Perkin Elmer) was used to measure fluorescence: set at 30°C with an excitation wavelength of 360 nm and emission wavelength of 460 nm.

Electrophoretic mobility shift assays

PCR products of ICE*Ml*Sym^{R7A} EMSA target DNA were amplified with primers containing a universal adaptor. Each PCR product was then subsequently amplified again using universal PCR primers "UNIVERSAL_IR800_FWD" and "UNIVERSAL_IR800_REV" (**Supplemental Table S2**), which each contained a 5'IRDye® 800 fluorescent label at the 5' end (IDT). Each EMSA binding reaction was made up to a total volume of 20 µL. Each reaction contained 10 µL of a DNA master mix (50 mM Tris-HCl, 150 mM NaCl, 5% *v/v* glycerol; pH 7.4, 1 unit of Poly[d(I-C)], 0.05 mg mL^{-1} sheared herring-sperm DNA, 0.1 mg mL^{-1} BSA, 0.5% *v/v* Tween20, 5 mM DTT and 10 nM 5'IRDye®-labelled PCR product) mixed with 10 µL of protein. Samples were incubated in the

dark for 30 min at ambient temperature. Two microlitres of orange loading-dye (0.3% *w/v* Orange-G, 65% *w/v* sucrose, 10 mM Tris pH 7.5, 10 mM EDTA) was added to each sample prior to loading onto a non-denaturing polyacrylamide gels contained 8% final *w/v* concentration of 29:1 acrylamide:bis-acrylamide (with 50 mM Tris pH 7.5, 380 mM glycine and 2 mM EDTA). Gels were pre-run for 1 hour at 80 V. Samples were electrophoresed at 80 V for ~3 h in 1x TGE buffer (50 mM Tris pH 7.5, 192 mM Glycine, 2 mM EDTA) at ambient temperature with gentle buffer agitation. EMSA gels were exposed on a ChemiDoc MP Imaging System (Bio-Rad, USA) using the "IRDye 800CW" setting with auto exposure setting 4x4.

Small angle X-ray scattering

All 6H-RdfS-*attP*_8 SAXS data were collected using size-exclusion chromatography-coupled synchrotron-SAXS (SEC-SY-SAXS) at the Australian Synchrotron SAX/WAXS beamline using a Superdex 200 Increase 5/150 GL column (GE Healthcare) controlled by a Shimadzu HPLC system. Protein and DNA samples were frozen and transported to the Australian synchrotron on dry ice in their experimental conditions. Concentrated 6H-RdfS (at ~10 mg mL^{-1}) was mixed with *attP*_8 40mer DNA (1 mM) at a 1:1 volume ratio and incubated at ambient temperature for 30 minutes before 50 µL was injected into the system. All experiments were performed in SAXS buffer (150 mM Tris-HCl, 300 mM NaCl, 5% *v/v* glycerol; pH 7.4). Refer to **Supplemental Table S3** for SAXS data-collection and analysis statistics. Scattering data were background-corrected using linear interpolation of the background from averaged frames using *scatterBrain* (Stephen Mudie, Australian Synchrotron). The intensity at zero ($I(0)$), Guinier range and radius of gyration (R_g) were determined using the *ATSAS* package (6) using *PRIMUS* (7) and *GNOM* (8). *Ab initio* dummy atom (bead) models were generated with *DAMMIF* (10 total models) (9) and filtered using *DAMAVER* (10). *CRYSOL* (11,12) was used to fit the molecular dynamics model of RdfS-*attP*_8 into the processed SAXS scattering, using default settings and constant subtraction.

Quantitative PCR

Buffered TY liquid seeder cultures (5 mL) were grown for 3 days until stationary phase, and sterile glycerol was added (15% *v/v*) prior to freezing at -80°C. TY cultures were then inoculated with 1/200 dilution of seeder culture and grown to late stationary phase (64 hours). DNA was extracted from stationary phase culture using PrepMan Ultra Sample Preparation Reagent (ThermoFisher). The percentage of cells within the population were quantified for RdfS/IntS-mediated excision.

Assays were performed on the ViiA 7 Real-Time PCR system (Applied Biosystems) with default settings and reactions carried out in 20 µL; 10 µL of SensiFAST Probe Lo-ROX Kit (Bioline), 250 nM of probe (carrying 3’ FAM fluorophore) and 900 nM each primer (IDT). Primer/probe amplification efficiencies were: *attP* (2.09), *attB* (2.04) and *melR* (2.03). Normalised results were calculated using the following equation described previously (1): $R_{att} = \frac{(E_{att})^{\Delta C_{t(att)}}}{(E_{melR})^{\Delta C_{t(melR)}}}$

Molecular modelling and simulation

The complex of the RdfS tetramer bound to *attP*_8 (referred to as RdfS-DNA in-text) was generated via molecular modelling in Schrodinger Suite 2019-4, adapting previous procedures for generating DNA-protein complexes (13). The RdfS structure (PDB 8DGL) was initially prepared using the Protein Preparation Wizard. The PDB was searched for proteins containing DNA-binding helix-turn-helix domains (InterPro IPR041657) with DNA bound, identifying one suitable template, that of the *Streptomyces* protein BldC (PDB 6AMA) (14); this structure was also prepared using the Protein Preparation Wizard. Alignment of the *attP*_8 sequence to the *smeA-ssfA* DNA sequence in PDB 6AMA was performed using the *needle* tool of EMBOSS (15), setting the gap open and gap extend penalties to their maxima. The DNA in PDB 6AMA was then trimmed to the 40-bp portion to which *attP*_8 best aligned as shown below:

*attP*_8	`1 -------GAATTTCGGCGCAGTTGGTCCAACGAGGCGGGGGGCTGGT--- 40`
smeA-ssfA‡	`1 ATTCGGGTAATTCGGGTAATTCGGGTAATTCGGGTAATTCGGGTAATTCG 50`
	`          ****: ** :   : ***    **:*  :   ** *::*`

†Exact matches are marked by asterisk (*); purine-purine and pyrimidine-pyrimidine matches are marked by colon (:). ‡Only the first half of this sequence is shown.

Each monomer of RdfS was then aligned to the relevant monomers of BldC interacting with the DNA. The resulting complex was then refined by performing Prime Side Chain Prediction and Prime Minimization on all residues. Following this, the DNA was then mutated in UCSF Chimera to the relevant sequence and the complex again refined by Prime Side Chain Prediction and Prime Minimization on all residues. To generate the N-terminally truncated $RdfS_{13-89}$, the first 12 amino acids were manually deleted from each monomer within PDB 8DGL and the refined RdfS-DNA complex.

The unbound forms of RdfS and the generated RdfS-DNA complexes were subject to molecular dynamics simulations. Parameterization was performed using AmberTools (16). Simulations were performed using GROMACS 2020.3 (17) patched with PLUMED 2.6.1 (18). RdfS was

parameterized with the AMBER *ff14SB* force field (19), while DNA was parameterized with the parmbsc0 parameter set (20) with ε/ζOL1 (21) and χOL4 (22) modifications. The resulting topologies were then ported to GROMACS format using *acpype* (23) and the remaining system setup completed in GROMACS. Structures were solvated in TIP3P water (24) in a dodecahedral box with a minimum of 10 Å distance from the protein/complex to the box edge. Unbound systems were charge neutralised with the addition of chloride ions, while bound systems were charge neutralised with the addition of sodium ions (25). Brief (0.1 ns each) equilibrations in the NVT and NPT ensembles were performed, with position restraints (1000 kJ/(mol nm^2)) on all heavy atoms, to achieve a target temperature of 310 K and a target pressure of 1 atm. The modified Berendsen thermostat (V-rescale) was employed for temperature coupling (26), and the Berendsen barostat was employed for pressure coupling (27). The smooth particle mesh Ewald method was employed for long-range electrostatics (28). All bonds were constrained using the LINCS algorithm (29). Coordinates were saved every 10 ps.

Following equilibration, the unbound forms of RdfS were subject to 50 ns of simulation. Root-mean-squared deviation (RMSD) analysis of trajectories was performed with respect to PDB 8DGL on the protein structures excluding the N-terminal region removed in the truncated $RdfS_{13-89}$. With respect to the RdfS-DNA complex, adiabatic-biased molecular dynamics (ABMD) was employed (30) following equilibration to generate a complex structure wherein the RdfS tetramer most closely recapitulated its superhelical conformation as observed in PDB 8DGL, followed by an unbiased simulation. ABMD was performed for 100 ns with a force constant of 0.0001 kJ/mol, followed by a 50 ns unbiased simulation. The collective variable biased was the distance in the atom-atom contact map from the desired target state; this was biased towards zero (i.e., towards an exact match to target state). The contact map considered pairs of atoms in the protein that moved together/apart by greater than 0.2 nm between the initially generated complex and PDB 8DGL and that occurred within a distance of r_0 of each other in either state. For the full length RdfS, r_0 was set to 0.4 nm, while for $RdfS_{13-89}$, r_0 was set to 0.6 nm. The distance of each atom-atom pair considered in the contact map was transformed using the following switching function:

$$s(r) = \frac{1 - \left(\frac{r}{r_0}\right)^6}{1 - \left(\frac{r}{r_0}\right)^{10}}$$

To prevent unwanted deformation of the complexes during the simulation, the following additional restraints were employed during the ABMD simulation:

- Harmonic restraints on the distance between atoms contributing to Watson-Crick hydrogen bonds between dT 19, dC 20, and dC 21 with their antisense base pairs, with a force constant of 10000 kJ/(mol nm^2) applied when the distances exceed 0.2 nm.
- Harmonic restraints on the distances between the centres of mass of the major groove-contacting helix of each RdfS monomer with each 10-bp segment of the DNA, with a force constant of 10000 kJ/(mol nm^2) applied to maintain these distances at their initial values.

For the full length RdfS, these restraints were employed during the first 2 ns of the ABMD simulation, then gradually decayed over the following 18 ns of the simulation. For $RdfS_{13\text{-}89}$, restraints were employed during the first 10 ns of the ABMD simulation, then gradually decayed over the following 40 ns of the simulation. Trajectories were clustered using the GROMOS algorithm (31) with a threshold of 0.2 nm, with the middle structure of the largest cluster selected as representative; the region selected for least squares fitting was the protein excluding the N-terminal removed in the truncated $RdfS_{13\text{-}89}$.

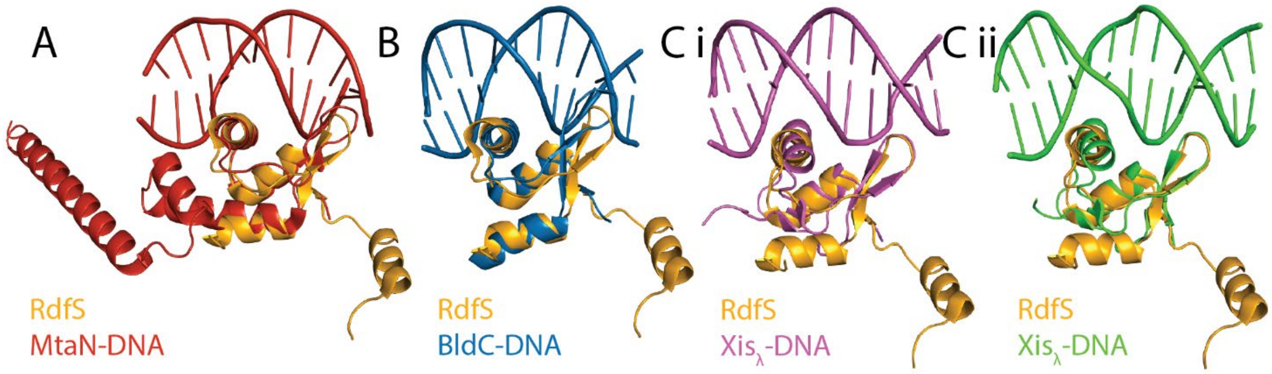

Supplemental Figure S1: Comparison of RdfS to other wHTH protein-DNA crystal structures. RdfS was superimposed onto each of the following wHTH proteins to visualise RdfS-DNA interactions: **(A)** MtaN (PDB 1R8D (32)), **(B)** BldC (PDB 6AMK (14)), **(C)** Xis (i) (PDB 2IEF (33)) and (ii) (PDB 1RH6 (34)). These models indicate RdfS likely binds DNA structurally-identically to most other wHTH proteins. These DNA regions were isolated and mapped to each chain monomer in the RdfS tetramer ASU shown in-text in **Figure 6A**.

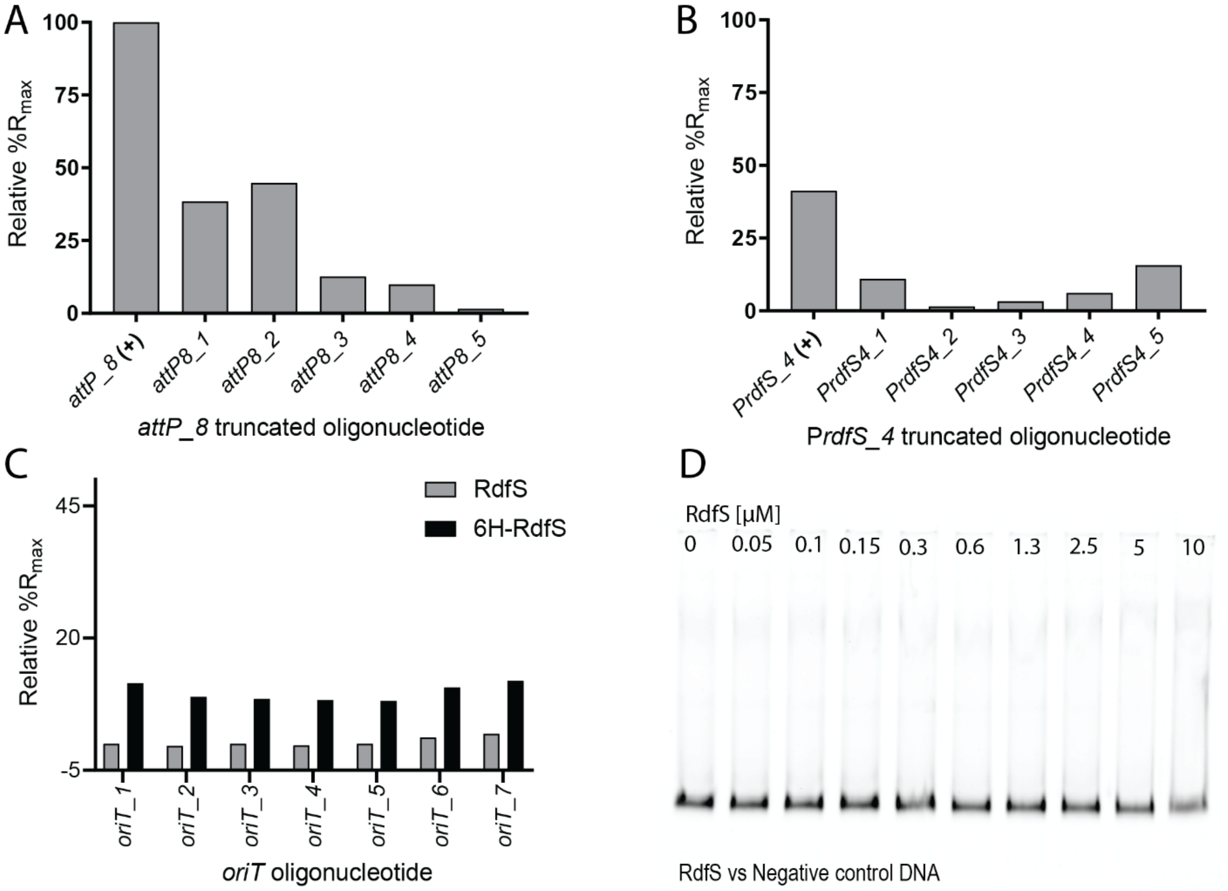

Supplemental Figure S2: DNA-binding of RdfS against various targets. 6H-RdfS/RdfS concentration for SPR DNA-footprinting in panels *(A)*, *(B)* and *(C)* were 1 µM. **(A)** SPR relative %R_{max} binding responses of truncated *attP*_8 oligonucleotides. The 40-bp *attP*_8 region was identified to be a binding target by RdfS, so further truncations of 20-bp (15-bp overlap) were screened for binding responses. No significant binding response (>50% R_{max} relative to 40-bp *attP*_8) was seen for any truncated region, suggesting the entire 40-bp region is required for efficient binding. **(B)** SPR relative %R_{max} binding responses of truncated P*rdfS*_4 oligonucleotides. Identical design to *attP*_8 oligomers: the full 40-bp P*rdfS*_4 oligonucleotide was truncated to 20-bp oligomers with 15-bp overlapping regions and were sequentially tested. No significant binding response was seen to any of the truncated P*rdfS*_4 oligonucleotides. **(C)** SPR RdfS vs *oriT*. To directly test for RdfS ability to regulate conjugation, we investigated the *oriT* DNA region as a potential target. No region of the *oriT* appeared to show a significant binding response to purified RdfS via SPR-based DNA-footprinting. **(D)** EMSA showing RdfS against a 100-bp non-specific fragment of DNA from the *intS* CDS. No shift is seen at any concentration, suggesting RdfS has a specificity for other EMSA target DNA shown in this study.

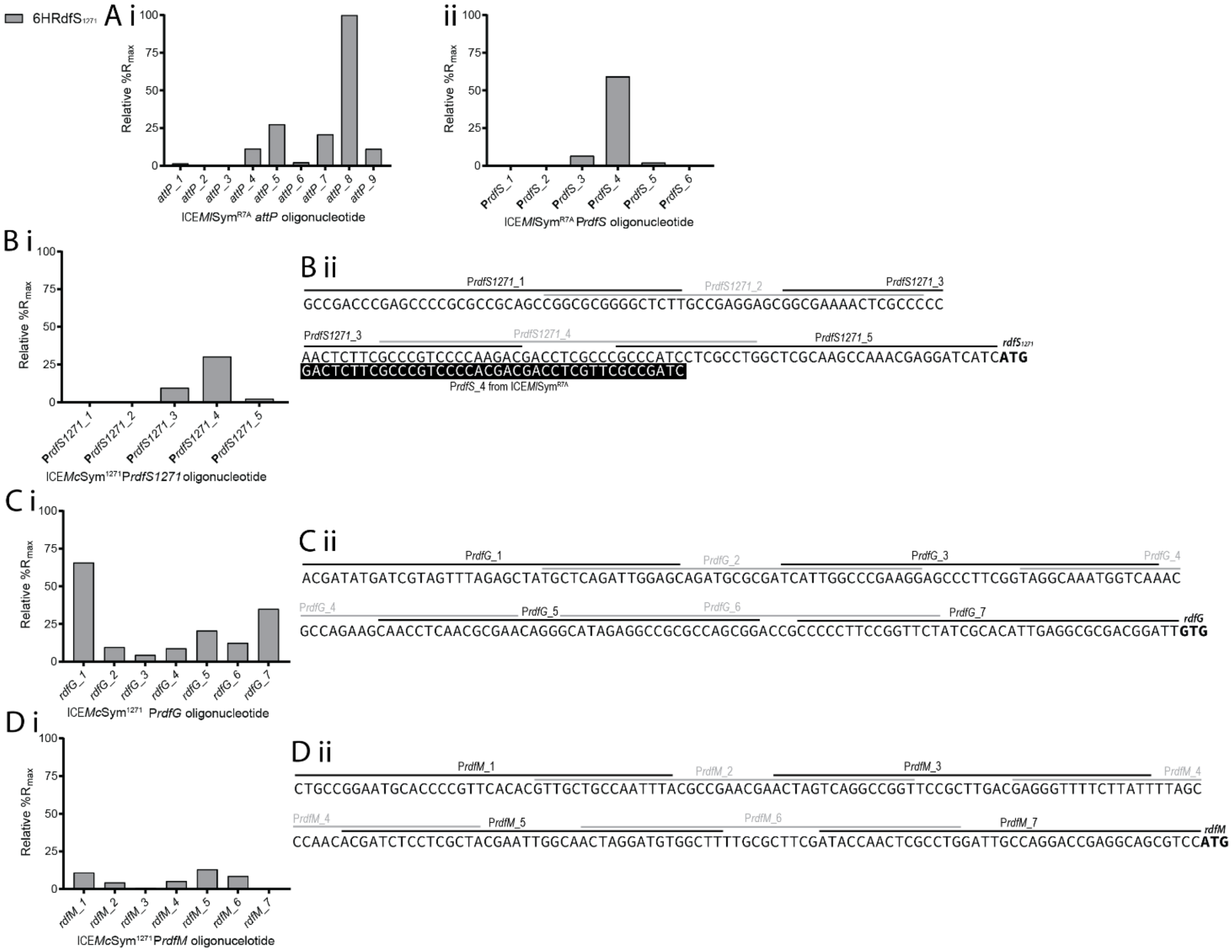

Supplemental Figure S3: Binding of WSM1271 RdfS using SPR DNA-footprinting. (A) Purified 6HRdfS1271 relative binding responses to the ICE*Ml*Sym[R7A] **(i)** *attP* region and **(ii)** P*rdfS* region. RdfS1271 appeared to have the same relative responses to these regions are previously described for 6H-RdfS/RdfS from ICE*Ml*Sym[R7A]. 6HRdfS1271 relative binding to **(i)** 40-bp oligonucleotides and **(ii)** accompanying promoter DNA array design of the: **(B)** ICE*Mc*Sym[1271] P*rdfS* region (P*rdfS1271*) (with the comparative location and sequence of ICE*Ml*Sym[R7A] P*rdfS*_4 shown in black), **(C)** P*rdfG* region and **(D)** P*rdfM* regions. These data are consistent with observations that RdfS1271 is a transcriptional activator of the other RDFs in ICE*Mc*Sym[1271].

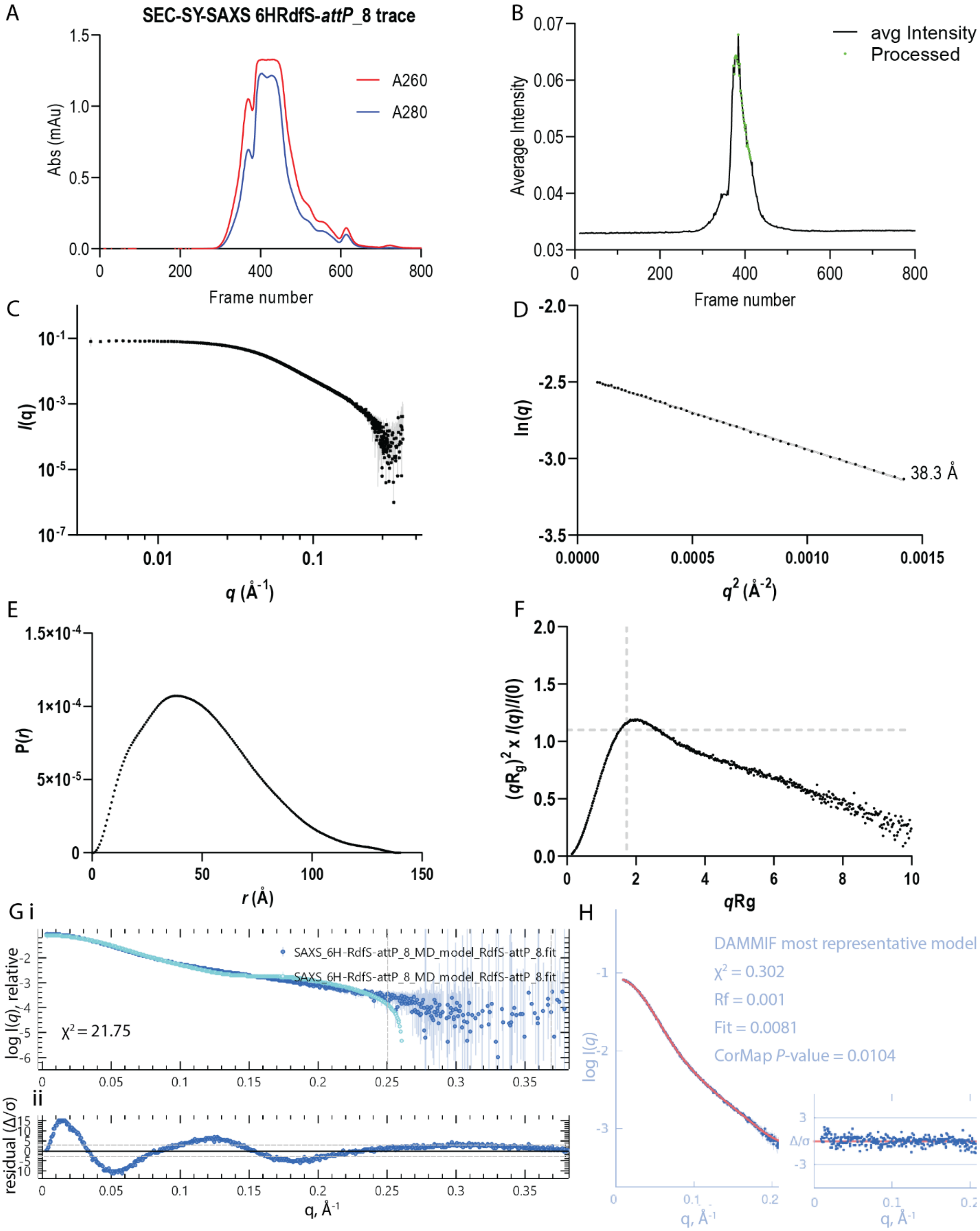

Supplemental Figure S4: SEC-SY-SAXS of 6H-RdfS-*attP*_8 complex. **(A)** UV elution profile showing absorbance at 260 (red) and 280 (blue) nm. Each frame represents 1 s of exposure **(B)** Scattering profile showing average scattering vs frame: frames 375 to 411 were processed in downstream analysis (shown as green points). **(C)** *I(q)* vs. *q* as log-linear plot. **(D)** Guinear plot for

the data, with the R_g shown next to the linear slope. **(E)** Distance-distribution P(*r*) vs *r* graph showing the D_{max} of ~138 Å (using R_g of 38.3). **(F)** Normalised (dimensionless) Kratky plot showing the elongation and order of the 6H-RdfS-*attP_8* complex with peaks beyond 1.104 and √3 shown as dashed lines. MW estimates from these data suggest tetramers (four copies) of 6H-RdfS binding the 40-bp *attP*_8 DNA region (see in-text). **(G)** CRYSOL fit of 6H-RdfS-*attP*_8 X-ray scattering to the molecular dynamics model of RdfS-DNA, reduced-chi squared value shown: **(i)** the fit of the model to the scattering and **(ii)** the residuals of the fit. **(H)** The fit and residuals for the "most representative" DAMMIF model of 6H-RdfS-*attP*_8 (as determined by DAMAVER) for the bead model shown in-text.

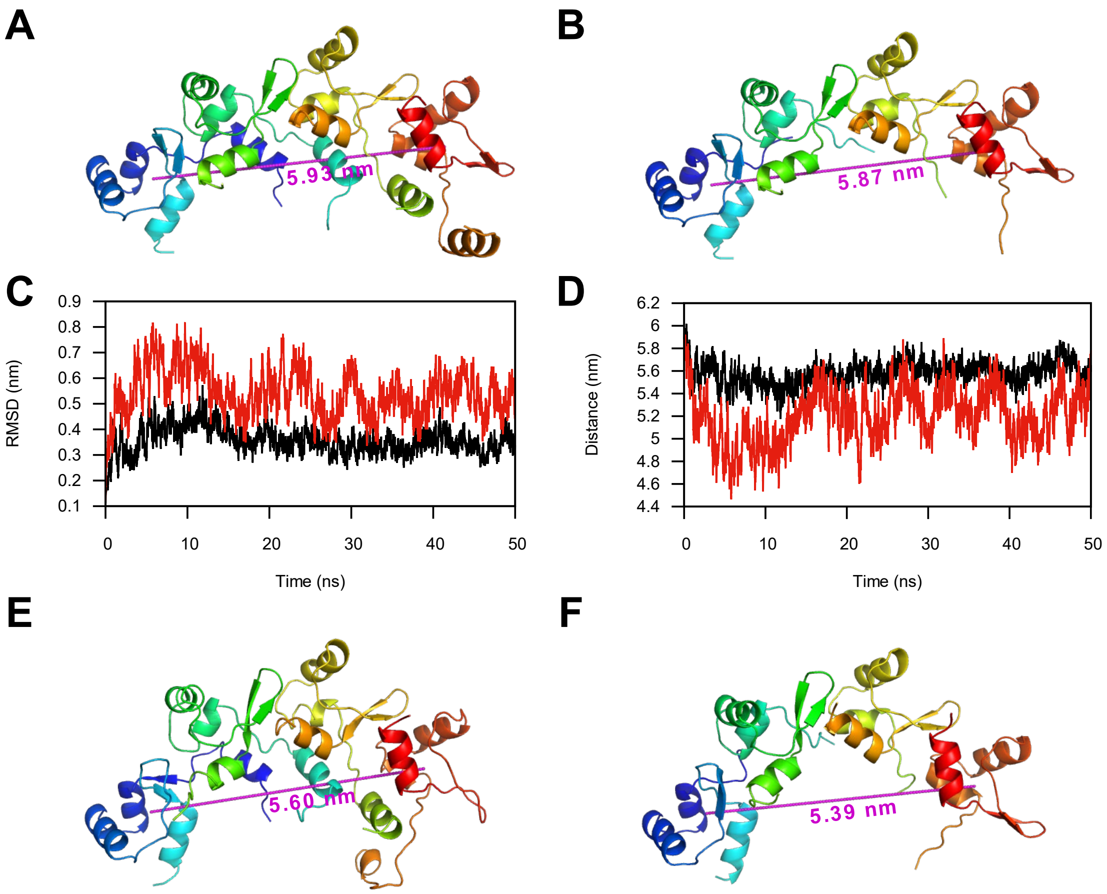

Supplemental Figure S5: Molecular dynamics simulation of apo full-length RdfS and N-terminally truncated RdfS$_{13\text{-}89}$. In all panels, the RdfS tetramer is coloured in a blue-to-red rainbow from N- to C-terminal, and the distance between the centres of mass of the capping monomers is shown as a pink line. **(A)** Structure of the unbound RdfS as determined by X-ray crystallography (PDB 8DGL). **(B)** Structure of unbound RdfS$_{13\text{-}89}$. **(C)** Root-mean-squared deviation over simulation trajectories for unbound full-length RdfS (black) and RdfS$_{13\text{-}89}$ (red), suggesting RdfS$_{13\text{-}89}$ is more flexible in simulation. **(D)** Distance between the centres of mass of capping monomers over simulation trajectories for unbound full-length RdfS (black) and RdfS$_{13\text{-}89}$ (red), with each model converging on a more compact overall-structure. **(E)** Representative structure of unbound full-length RdfS obtained from molecular dynamics simulation. **(F)** Representative structure of unbound RdfS$_{13\text{-}89}$ obtained from molecular dynamics simulation.

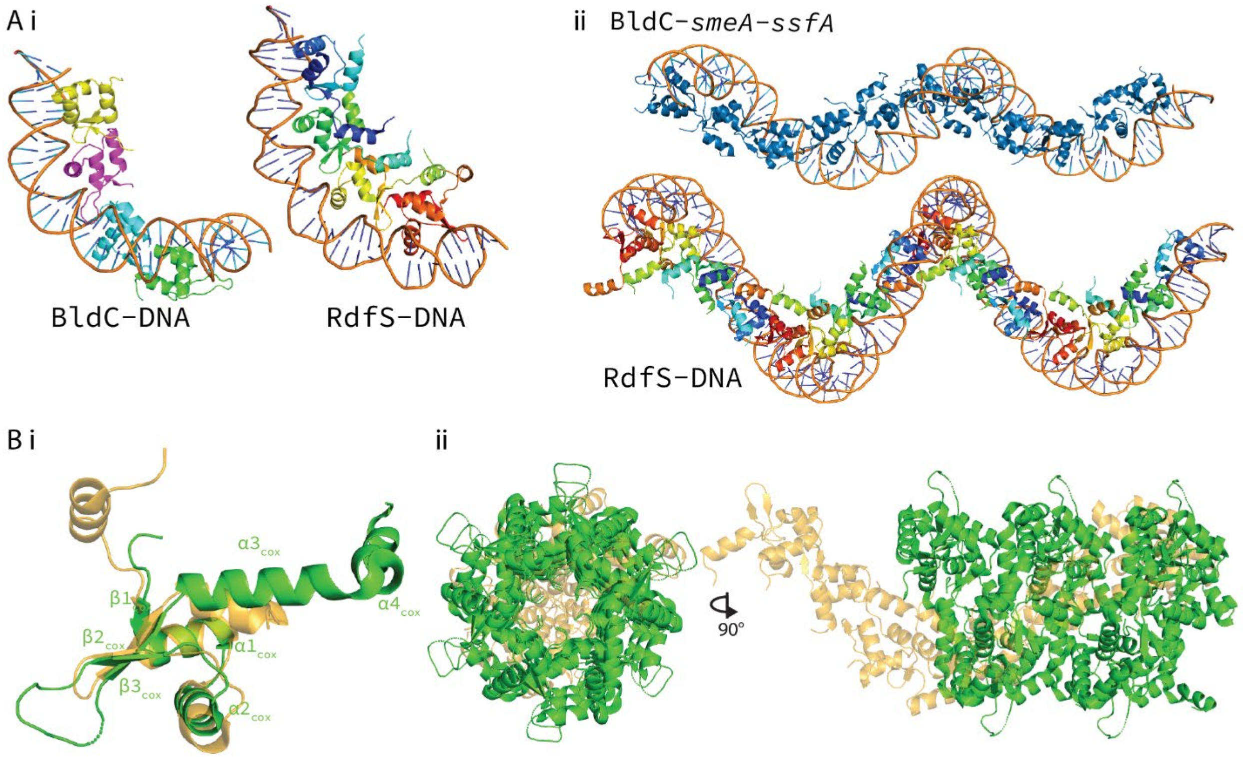

Supplemental Figure S6: Comparisons of BldC-DNA complex with RdfS-DNA complex model and P2 Cox structural similarity. (A)(i) Visualisation of DNA-morphology for BldC-DNA 40mer (left) and RdfS-DNA 40mer (right) based on BldC structure (PDB 6AMA (14)) and representative MD model of RdfS generated in this study. **(ii)** Extended filament of BldC-*smeA-ssfA* DNA (top) and RdfS-DNA (bottom) showing the enhanced, more pronounced lengthening of helical coils from our RdfS-DNA MD models. **(B)** P2 Cox (PDB 4LHF) shown in green, RdfS shown in yellow. **(i)** Key secondary structures for P2 Cox labelled. The $\alpha3_{cox}$ and $\alpha4_{cox}$ helices enable a left-handed spiral helical quaternary structure – with 55% of the monomeric surface interacting with adjacent protomers in crystal (35) – in comparison to only ~30% for RdfS protomer-interfaces. **(ii)** Comparison between RdfS and P2 Cox superhelix. P2 Cox additionally forms an apo helix in crystal, although significantly tighter between protomers per turn, mimicking a denser structure.

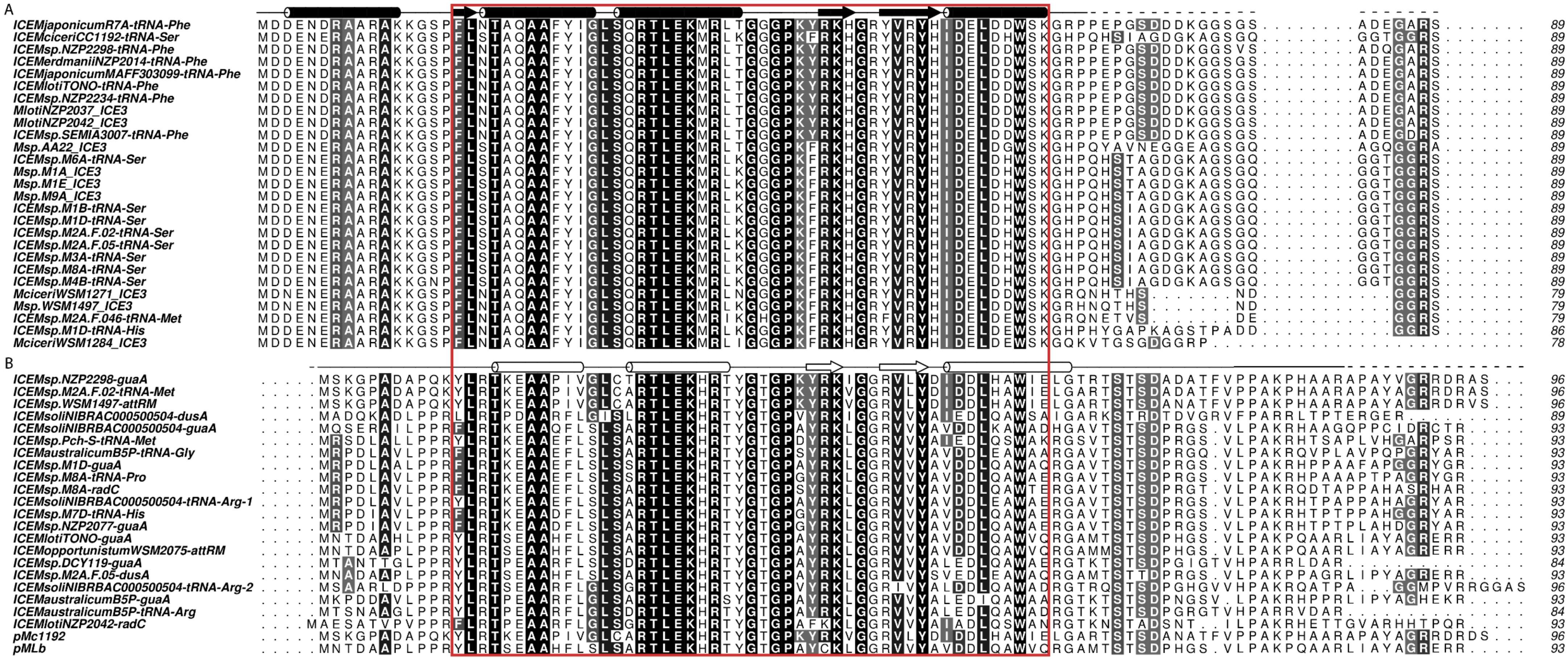

Supplemental Figure S7: Alignment of RdfS homologues. **(A)** RdfS encoded in ICESym family. ICE*Ml*Sym^{R7A} secondary structure shown at top (black). **(B)** Other RdfS from *Mesorhizobium* ICEs and plasmids, with predicted secondary structure shown in white. Each RdfS is named from the corresponding ICE or plasmid according to the nomenclature developed by Colombi *et al.* (36). Alignment generated using Clustal Omega (37) and visualised in Aline (38). Residue similarity is coloured from least to most (white> grey> black). The predicted wHTH region (based on ICE*Ml*Sym^{R7A} RdfS) is shown as a red box.

Supplemental Table S1. Strains and plasmids

Strain	Description	Source
Escherichia coli		
EPI300	F^{-} *mcrA* Δ(*mcrCB-hsdSMR-mrr*) (Str^{R}) Φ80d*lacZ*ΔM15 Δ*lacX74 rec*A1 *end*A1 *araD*139 Δ(*ara*, *leu*)7697*galU galK* λ^{-} *rpsL nupG trfA ton*A *dhfr*	Epicentre
ST18	S17 λ*pir* Δ*hemA*	(39)
BL21(DE3)pLysS	F^{-} *ompT gal dcm lon* hsdSB(*rB*$^{-}$ *mB*$^{-}$) λ(DE3) pLysS, Cm^{R}	(40)
NiCo21(DE3)	Modified BL21(DE3) with reduced endogenous nickel-binding proteins	(41)
Mesorhizobium japonicum		
R7A	Wild-type symbiotic strain; field re-isolate ICMP 3153	(42)
R7ANS	Non-symbiotic derivative of R7A; lacks ICE*Ml*Sym^{R7A}	(1)
R7AΔ*rdfS*	Δ*rdfS* markerless in-frame deletion in R7A	(1)
R7AΔ*qseM*	Δ*qseM* markerless in-frame deletion in R7A	(4)
R7AΔ*qseM*Δ*rdfS*	*rdfS* markerless in-frame deletion in R7AΔ*qseM* (4) constructed using pJK301 (1)	This study
R7ANS::pFUS2P1-P5	pFUS2P1-P5 integrated into R7ANS *phe*-tRNA gene. Contains 5' end of P*intS* fused with *lacZ.*	(43)
**Plasmids**		
pETM-11	N-terminally tagged 6xhistidine protein expression vector, T7-*lac*, Km^{R}	(44)
pETM-RdfS	pETM-11 with ICE*Ml*Sym^{R7A} RdfS cloned into NcoI/BamHI sites	(45)
pETM-RdfS13-89	pETM-11 with ICE*Ml*Sym^{R7A} RdfS 13-89 cloned into NcoI/BamHI sites	This study
pETM-RdfS1271	pETM-11 with ICE*Mc*Sym^{1271} RdfS cloned into NcoI/BamHI sites	This study
pSRKKm	pBBR1MCS-2-derived broad-host-range expression vector containing *lac* promoter and *lacI*^{q}*, lacZα*. Nm/Km^{R}.	(46)
pSRKrdfS	pSRKKm containing 270-bp *rdfS* gene cloned downstream of *lac* promoter. Nm^{R}.	(43)
pFAJ1700	Broad-host-range plasmid, *oriV*RK2 Tc^{R}, Ap^{R}	(47)
pFAJ1708	Broad-host-range cosmid containing *nptII* promoter; λ cos site, *oriT*. Tc^{R}	(47)
pJJ611	pFAJ1708 containing *intS* coding sequence and upstream region.	(1)
pJR202	pFAJ1700 containing *rdfS* and P*rdfS* promoter region.	(1)
pFJrdfS13-89	pFAJ1700 containing *rdfS*$_{13\text{-}89}$ cloned downstream of P*rdfS*	This study
pFJrdfS1-64	pFAJ1700 containing *rdfS*$_{1\text{-}64}$ cloned downstream of P*rdfS*	This study
pF08rdfS13-89	pFAJ1708 containing *rdfS*$_{13\text{-}89}$ cloned downstream of *nptII*	This study

pSDz	Derivative of pFAJ1700, carrying *lacZ* from pFUS2 and *lacI*-*lacO* from pSRKKm, Ap^{R}, Tc^{R}	(48)
pSDRdfS-lacZ	pSDZ carrying the *rdfS* promoter region cloned upstream of *lacZ*	(48)
pFseArdfS-lacZ	pSDRdfS-lacZ carrying *fseA* CDS cloned downstream of *lacI*	(48)
pFUS2	Suicide vector, *oriC*^{ColE1} *oriT*^{RK2} *lacZ* transcriptional reporter. Gm^{R}.	(49)
pFUS2P1-P5	Modified pFUS2 vector containing 287-bp ICE*Ml*Sym^{R7A} *attP* “arm” P regions 1-5.	(43)
pPR3	pVS1/p15a replicons, carries *nptII* promoter, Km^{R}.	(50)
pPROBE-GT	pVS1/p15a replicons, Gm^{R}	(51)
pPR3G	pPROBE-GT carrying the *nptII* promoter amplified from pFAJ1708. Gm^{R}	(52)

Supplemental Table S2. Oligonucleotides used in this study

Oligonucleotide	Sequence	Description
**Primers**		
N_rdfS_NcoI_2	AATTCCATGGGCATGAAGGGCAGTCCGTTTCTGAA	PCR amplification of *rdfS* residues 13-89.
RdfS_HindIII_R	ATATGGATCCTTATCATGAGCGGGCTCCCTCG	PCR amplification of *rdfS* residues 13-89.
109CN3-PstI	AATACTGCAGGATCAGATGGATGGAAGGCCGCTGGCTCAT	PCR 3' primer for *rdfS* truncation constructs in pFAJ1700/pFAJ1708 backbones
RdfSnative5'BamHI_new	AAGATGGATCCTGACCGGCCGGCTCAAGCCCT	PCR amplification for *rdfS* full-length in pFAJ1700/pFAJ1708 backbones BamHI/PstI sites
109CN5-bamhi	AAGATGGATCCTGACTAGCTAACGAGGACCATCATGGACGA	PCR amplification for *rdfS*$_{1-64}$ in pFAJ1708/pFAJ1700 backbones BamHI/PstI sites
109CN5-bamHI-N-trunc	AAGATGGATCCTGACTAGCTAACGAGGACCATCATGAAGG	PCR amplification for *rdfS*$_{13-89}$ in pFAJ1700/pFAJ1708 backbones BamHI/PstI sites
1271_RdfS_F_NcoI	ATATCCATGGACAACGAAAACGAACGCG	PCR amplification of WSM1271 RdfS.
1271_RdfS_R_HindIII	ATATAAGCTTTTATCATGAGCGGCCTCCATCG	PCR amplification of WSM1271 RdfS.
attP_R7A_UniIR_FWD	GGTTGAATGCTCGACACTAGTTGGTACTTCTGTTGGCAC	Amplify the R7A *attP* P1-P5 region with universal IR800 tag for EMSA
attP_R7A_UniIR_REV	GAACTAGTAGCGCACGCTGGATACCATCAGCATCTCCAA	Amplify the R7A *attP* P1-P5 region with universal IR800 tag for EMSA
IR800_rdfSpro_F	GGTTGAATGCTCGACACTACCGGCCGGCTCAAGCCCTCG	Amplify R7A *rdfS* promoter (entire intergenic region of msi110-rdfS) with universal IR800 tag for IR800 labelling EMSAs
IR800_rdfSpro_R	GAACTAGTAGCGCACGCTGGATGGTCCTCGTTTCGCTTCGG	Amplify R7A *rdfS* promoter (entire intergenic region of msi110-rdfS) with universal IR800 tag for IR800 labelling EMSAs
IR800_oriT_F	GGTTGAATGCTCGACACTAACCAGCCTCCGGGCAAATTCAA	Amplify R7A *oriT* region (entire intergenic region of msi107-rlxS) with universal IR800 tag for IR800 labelling EMSAs
IR800_oriT_R	GAACTAGTAGCGCACGCTGGATGCCCTCAACATCGCTAG	Amplify R7A *oriT* region (entire intergenic region of msi107-rlxS) with universal IR800 tag for IR800 labelling EMSAs
IR800_NEG_F_intSmid	AATGCTCGACACTACCTATCGTTTTGGCG	Amplify the middle of R7A IntS with universal IR800 universal sequence adaptors for use in IR800 EMSAs as negative control.
IR800_NEG_R_intSmid	TAGTAGCGCACGCTGGATAGCTGCCCAAC	Amplify the middle of R7A IntS with universal IR800 universal sequence adaptors for use in IR800 EMSAs as negative control.

UNIVERSAL_IR800_FWD	/5IRD800/TACGTGGTTGAATGCT CGACACTA	Primers that amplify universal sequence and tag with 5' IRDye® 800CW (N-hydroxysuccinimide ester) FWD.
UNIVERSAL_IR800_REV	/5IRD800/TACGGGAACTAGTAGC GCCGCTG	Primers that amplify universal sequence and tag with 5' IRDye® 800CW (N-hydroxysuccinimide ester) REV.
attP_8_40bp_F	GAATTTCGGCGCAGTTGGTCCA ACGAGGCGGGGGGCTGGT	40-bp *attP* oligonucleotide used in analytical SEC and SEC-SY-SAXS
attP_8_40bp_R	ACCAGCCCCCCGCCTCGTTGGA CCAACTGCGCCGAAATTC	40-bp *attP* oligonucleotide used in analytical SEC and SEC-SY-SAXS
**Surface Plasmon Resonance Oligonucleotides**$^{\neq}$		
ReDCaT_F_bio	/5BIOSG/GCAGGAGGACGTAGGGTAGG	
ReDCaT_COMP	CCTACCCTACGTCCTCCTGC	
attP_1_For	GTTGGTACTTCTGTTGGCACGCAGGGACGATGTTGGAGGC	
attP_1_Rev	GCCTCCAACATCGTCCCTGCGTGCCAACAGAAGTACCAACcctaccctacgtcctcctgc	
attP_2_For	TGTTGGAGGCCAAACATTTGTAAACAAAGTCAAATTTGTT	
attP_2_Rev	AACAAATTTGACTTTGTTTACAAATGTTTGGCCTCCAACAcctaccctacgtcctcctgc	
attP_3_For	CAAATTTGTTTCTCTTGGATTCCGCCTCTGGGCACCATCC	
attP_3_Rev	GGATGGTGCCCAGAGGCGGAATCCAAGAGAAACAAATTTGcctaccctacgtcctcctgc	
attP_4_For	GGCACCATCCCCTCTTCTCCCGACAGTCTCTTTCGCCGAA	
attP_4_Rev	TTCGGCGAAAGAGACTGTCGGGAGAAGAGGGGATGGTGCCcctaccctacgtcctcctgc	
attP_5_For	TTTCGCCGAAGCCTCTACGCTTCTGTCTCGGCAACTAGCT	
attP_5_Rev	AGCTAGTTGCCGAGACAGAAGCGTAGAGGCTTCGGCGAAAcctaccctacgtcctcctgc	
attP_6_For	GCAACTAGCTGAAATCCTTACTGATTTCGGCGCGCAAAGC	
attP_6_Rev	GCTTTGCGCGCCGAAATCAGTAAGGATTTCAGCTAGTTGCcctaccctacgtcctcctgc	
attP_7_For	CGCGCAAAGCGCGGATGGTCGGGTTTTGTGGAATTTCGGC	
attP_7_Rev	GCCGAAATTCCACAAAACCCGACCATCCGCGCTTTGCGCGcctaccctacgtcctcctgc	
attP_8_For	GAATTTCGGCGCAGTTGGTCCAACGAGGCGGGGGGCTGGT	
attP_8_Rev	ACCAGCCCCCCGCCTCGTTGGACCAACTGCGCCGAAATTCcctaccctacgtcctcctgc	
attP_9_For	GGGGGCTGGTATTTTCGGTGGTATTGGAGATGCTGATGGT	
attP_9_Rev	ACCATCAGCATCTCCAATACCACCGAAAATACCAGCCCCCcctaccctacgtcctcctgc	
oriT_1_For	ACCAGCCTCCGGGCAAATTCAAGCGAAACCTGAGTCTCGC	
oriT_1_Rev	GCGAGACTCAGGTTTCGCTTGAATTTGCCCGGAGGCTGGTcctaccctacgtcctcctgc	
oriT_2_For	AAACCTGAGTCTCGCTGGCCCACCCTCCTGGCGCCGCCGG	
oriT_2_Rev	CCGGCGGCGCCAGGAGGGTGGGCCAGCGAGACTCAGGTTTcctaccctacgtcctcctgc	
oriT_3_For	TCCTGGCGCCGCCGGCAGCCTCCGGCAAAAACGATGTGCA	
oriT_3_Rev	TGCACATCGTTTTTGCCGGAGGCTGCCGGCGGCGCCAGGAcctaccctacgtcctcctgc	
oriT_4_For	CAAAAACGATGTGCAGACAAAGGCTTCGCCGCCTCATGGA	
oriT_4_Rev	TCCATGAGGCGGCGAAGCCTTTGTCTGCACATCGTTTTTGcctaccctacgtcctcctgc	
oriT_5_For	TCGCCGCCTCATGGAGGCGGTGCCTTTATCTTGCCTTACG	
oriT_5_Rev	CGTAAGGCAAGATAAAGGCACCGCCTCCATGAGGCGGCGAcctaccctacgtcctcctgc	
oriT_6_For	TTATCTTGCCTTACGCGCCCCTCGCCTTAAGCGTCCCTGG	

oriT_6_Rev	CCAGGGACGCTTAAGGCGAGGGGCGCGTAAGGCAAGATAAcctaccctacgtcctcctgc
oriT_7_For	CTTAAGCGTCCCTGGCCCCTCCCGGATGCCCTCAACATCG
oriT_7_Rev	CGATGTTGAGGGCATCCGGGAGGGGCCAGGGACGCTTAAGcctaccctacgtcctcctgc
PrdfS_1_For	CAAGCCCTCGCGCCGCAACTCGGCGCGGGGGTTCCCGCCG
PrdfS_1_Rev	CGGCGGGAACCCCCGCGCCGAGTTGCGGCGCGAGGGCTTGcctaccctacgtcctcctgc
PrdfS_2_For	CGGGGGTTCCCGCCGACGCTCGCCCGAAGGGGCGGCGAAA
PrdfS_2_Rev	TTTCGCCGCCCCTTCGGGCGAGCGTCGGCGGGAACCCCCGcctaccctacgtcctcctgc
PrdfS_3_For	GAAGGGGCGGCGAAAACTCCCACCCGACTCTTCGCCCGTC
PrdfS_3_Rev	GACGGGCGAAGAGTCGGGTGGGAGTTTTCGCCGCCCCTTCcctaccctacgtcctcctgc
PrdfS_4_For	GACTCTTCGCCCGTCCCCACGACGACCTCGTTCGCCGATC
PrdfS_4_Rev	GATCGGCGAACGAGGTCGTCGTGGGGACGGGCGAAGAGTCcctaccctacgtcctcctgc
PrdfS_5_For	CCTCGTTCGCCGATCCTCGCGGTTGCTTGTCCGAAGCGAA
PrdfS_5_Rev	TTCGCTTCGGACAAGCAACCGCGAGGATCGGCGAACGAGGcctaccctacgtcctcctgc
PrdfS_5_For	ATCCTCGCGGTTGCTTGTCCGAAGCGAAACGAGGACCATC
PrdfS_5_Rev	GATGGTCCTCGTTTCGCTTCGGACAAGCAACCGCGAGGATcctaccctacgtcctcctgc
PrdfG_1_For	ACGATATGATCGTAGTTTAGAGCTATGCTCAGATTGGAGC
PrdfG_1_Rev	GCTCCAATCTGAGCATAGCTCTAAACTACGATCATATCGTcctaccctacgtcctcctgc
PrdfG_2_For	TGCTCAGATTGGAGCAGATGCGCGATCATTGGCCCGAAGG
PrdfG_2_Rev	CCTTCGGGCCAATGATCGCGCATCTGCTCCAATCTGAGCAcctaccctacgtcctcctgc
PrdfG_3_For	TCATTGGCCCGAAGGAGCCCTTCGGTAGGCAAATGGTCAA
PrdfG_3_Rev	TTGACCATTTGCCTACCGAAGGGCTCCTTCGGGCCAATGAcctaccctacgtcctcctgc
PrdfG_4_For	TAGGCAAATGGTCAAACGCCAGAAGCAACCTCAACGCGAA
PrdfG_4_Rev	TTCGCGTTGAGGTTGCTTCTGGCGTTTGACCATTTGCCTAcctaccctacgtcctcctgc
PrdfG_5_For	CAACCTCAACGCGAAAGTTGCCAGGGCATAGAGGCCGCGC
PrdfG_5_Rev	GCGCGGCCTCTATGCCCTGGCAACTTTCGCGTTGAGGTTGcctaccctacgtcctcctgc
PrdfG_6_For	GCATAGAGGCCGCGCCAGCGGACCGCCCCCTTCCGGTTCT
PrdfG_6_Rev	AGAACCGGAAGGGGGCGGTCCGCTGGCGCGGCCTCTATGccctaccctacgtcctcctgc
PrdfG_7_For	CCCCCTTCCGGTTCTATCGCACATTGAGGCGCGACGGATT
PrdfG_7_Rev	AATCCGTCGCGCCTCAATGTGCGATAGAACCGGAAGGGGGcctaccctacgtcctcctgc
PrdfM_1_For	CTGCCGGAATGCACCCCGTTCACACGTTGCTGCCAATTTA
PrdfM_1_Rev	TAAATTGGCAGCAACGTGTGAACGGGGTGCATTCCGGCAGcctaccctacgtcctcctgc
PrdfM_2_For	GTTGCTGCCAATTTACGCCGAACGAACTAGTCAGGCCGGT
PrdfM_2_Rev	ACCGGCCTGACTAGTTCGTTCGGCGTAAATTGGCAGCAACcctaccctacgtcctcctgc
PrdfM_3_For	ACTAGTCAGGCCGGTTCCGCTTGACGAGGGTTTTCTTATT
PrdfM_3_Rev	AATAAGAAAACCCTCGTCAAGCGGAACCGGCCTGACTAGTcctaccctacgtcctcctgc
PrdfM_4_For	GAGGGTTTTCTTATTTTAGCCCAACACGATCTCCTCGCTA
PrdfM_4_Rev	TAGCGAGGAGATCGTGTTGGGCTAAAATAAGAAAACCCTCcctaccctacgtcctcctgc
PrdfM_5_For	ACGATCTCCTCGCTACGAATTGGCAACTAGGATGTGGCTT
PrdfM_5_Rev	AAGCCACATCCTAGTTGCCAATTCGTAGCGAGGAGATCGTcctaccctacgtcctcctgc
PrdfM_6_For	ACTAGGATGTGGCTTTTGCGCTTCGATACCAACTCGCCTG
PrdfM_6_Rev	CAGGCGAGTTGGTATCGAAGCGCAAAAGCCACATCCTAGTcctaccctacgtcctcctgc

PrdfM_7_For	ATACCAACTCGCCTGGATTGCCAGGACCGAGGCAGCGTCC
PrdfM_7_Rev	GGACGCTGCCTCGGTCCTGGCAATCCAGGCGAGTTGGTATcctaccctacgtcctcctgc
PrdfS1271_1_For	GCCGACCCGAGCCCCGCGCCGCAGCCGGCGCGGGGCTCTT
PrdfS1271_1_Rev	AAGAGCCCCGCGCCGGCTGCGGCGCGGGGCTCGGGTCGGCcctaccctacgtcctcctgc
PrdfS1271_2_For	CGGCGCGGGGCTCTTGCCGAGGAGCGGCGAAAACTCGCCC
PrdfS1271_2_Rev	GGGCGAGTTTTCGCCGCTCCTCGGCAAGAGCCCCGCGCCGcctaccctacgtcctcctgc
PrdfS1271_3_For	GGCGAAAACTCGCCCCCAACTCTTCGCCCGTCCCCAAGAC
PrdfS1271_3_Rev	GTCTTGGGGACGGGCGAAGAGTTGGGGGCGAGTTTTCGCCcctaccctacgtcctcctgc
PrdfS1271_4_For	GCCCGTCCCCAAGACGACCTCGCCCGCCCATCCTCGCCTG
PrdfS1271_4_Rev	CAGGCGAGGATGGGCGGGCGAGGTCGTCTTGGGGACGGGCcctaccctacgtcctcctgc
PrdfS1271_5_For	GCCCATCCTCGCCTGGCTCGCAAGCCAAACGAGGATCATC
PrdfS1271_5_Rev	GATGATCCTCGTTTGGCTTGCGAGCCAGGCGAGGATGGGCcctaccctacgtcctcctgc
attP_8_M01_F	CAATTTCGGCGCAGTTGGTCCAACGAGGCGGGGGGCTGGT
attP_8_M01_R	ACCAGCCCCCCGCCTCGTTGGACCAACTGCGCCGAAATTGcctaccctacgtcctcctgc
attP_8_M02_F	GTATTTCGGCGCAGTTGGTCCAACGAGGCGGGGGGCTGGT
attP_8_M02_R	ACCAGCCCCCCGCCTCGTTGGACCAACTGCGCCGAAATACcctaccctacgtcctcctgc
attP_8_M03_F	GATTTTCGGCGCAGTTGGTCCAACGAGGCGGGGGGCTGGT
attP_8_M03_R	ACCAGCCCCCCGCCTCGTTGGACCAACTGCGCCGAAAATCcctaccctacgtcctcctgc
attP_8_M04_F	GAAATTCGGCGCAGTTGGTCCAACGAGGCGGGGGGCTGGT
attP_8_M04_R	ACCAGCCCCCCGCCTCGTTGGACCAACTGCGCCGAATTTCcctaccctacgtcctcctgc
attP_8_M05_F	GAATATCGGCGCAGTTGGTCCAACGAGGCGGGGGGCTGGT
attP_8_M05_R	ACCAGCCCCCCGCCTCGTTGGACCAACTGCGCCGATATTCcctaccctacgtcctcctgc
attP_8_M06_F	GAATTACGGCGCAGTTGGTCCAACGAGGCGGGGGGCTGGT
attP_8_M06_R	ACCAGCCCCCCGCCTCGTTGGACCAACTGCGCCGTAATTCcctaccctacgtcctcctgc
attP_8_M07_F	GAATTTGGGCGCAGTTGGTCCAACGAGGCGGGGGGCTGGT
attP_8_M07_R	ACCAGCCCCCCGCCTCGTTGGACCAACTGCGCCCAAATTCcctaccctacgtcctcctgc
attP_8_M08_F	GAATTTCCGCGCAGTTGGTCCAACGAGGCGGGGGGCTGGT
attP_8_M08_R	ACCAGCCCCCCGCCTCGTTGGACCAACTGCGCGGAAATTCcctaccctacgtcctcctgc
attP_8_M09_F	GAATTTCGCCGCAGTTGGTCCAACGAGGCGGGGGGCTGGT
attP_8_M09_R	ACCAGCCCCCCGCCTCGTTGGACCAACTGCGGCGAAATTCcctaccctacgtcctcctgc
attP_8_M10_F	GAATTTCGGGGCAGTTGGTCCAACGAGGCGGGGGGCTGGT
attP_8_M10_R	ACCAGCCCCCCGCCTCGTTGGACCAACTGCCCCGAAATTCcctaccctacgtcctcctgc
attP_8_M11_F	GAATTTCGGCCCAGTTGGTCCAACGAGGCGGGGGGCTGGT
attP_8_M11_R	ACCAGCCCCCCGCCTCGTTGGACCAACTGGGCCGAAATTCcctaccctacgtcctcctgc
attP_8_M12_F	GAATTTCGGCGGAGTTGGTCCAACGAGGCGGGGGGCTGGT
attP_8_M12_R	ACCAGCCCCCCGCCTCGTTGGACCAACTCCGCCGAAATTCcctaccctacgtcctcctgc
attP_8_M13_F	GAATTTCGGCGCTGTTGGTCCAACGAGGCGGGGGGCTGGT
attP_8_M13_R	ACCAGCCCCCCGCCTCGTTGGACCAACAGCGCCGAAATTCcctaccctacgtcctcctgc
attP_8_M14_F	GAATTTCGGCGCACTTGGTCCAACGAGGCGGGGGGCTGGT
attP_8_M14_R	ACCAGCCCCCCGCCTCGTTGGACCAAGTGCGCCGAAATTCcctaccctacgtcctcctgc
attP_8_M15_F	GAATTTCGGCGCAGATGGTCCAACGAGGCGGGGGGCTGGT

attP_8_M15_R	ACCAGCCCCCCGCCTCGTTGGACCATCTGCGCCGAAATTCcctaccctacgtcctcctgc
attP_8_M16_F	GAATTTCGGCGCAGTAGGTCCAACGAGGCGGGGGGCTGGT
attP_8_M16_R	ACCAGCCCCCCGCCTCGTTGGACCTACTGCGCCGAAATTCcctaccctacgtcctcctgc
attP_8_M17_F	GAATTTCGGCGCAGTTCGTCCAACGAGGCGGGGGGCTGGT
attP_8_M17_R	ACCAGCCCCCCGCCTCGTTGGACGAACTGCGCCGAAATTCcctaccctacgtcctcctgc
attP_8_M18_F	GAATTTCGGCGCAGTTGCTCCAACGAGGCGGGGGGCTGGT
attP_8_M18_R	ACCAGCCCCCCGCCTCGTTGGAGCAACTGCGCCGAAATTCcctaccctacgtcctcctgc
attP_8_M19_F	GAATTTCGGCGCAGTTGGACCAACGAGGCGGGGGGCTGGT
attP_8_M19_R	ACCAGCCCCCCGCCTCGTTGGTCCAACTGCGCCGAAATTCcctaccctacgtcctcctgc
attP_8_M20_F	GAATTTCGGCGCAGTTGGTGCAACGAGGCGGGGGGCTGGT
attP_8_M20_R	ACCAGCCCCCCGCCTCGTTGCACCAACTGCGCCGAAATTCcctaccctacgtcctcctgc
attP_8_M21_F	GAATTTCGGCGCAGTTGGTCGAACGAGGCGGGGGGCTGGT
attP_8_M21_R	ACCAGCCCCCCGCCTCGTTCGACCAACTGCGCCGAAATTCcctaccctacgtcctcctgc
attP_8_M22_F	GAATTTCGGCGCAGTTGGTCCTACGAGGCGGGGGGCTGGT
attP_8_M22_R	ACCAGCCCCCCGCCTCGTAGGACCAACTGCGCCGAAATTCcctaccctacgtcctcctgc
attP_8_M23_F	GAATTTCGGCGCAGTTGGTCCATCGAGGCGGGGGGCTGGT
attP_8_M23_R	ACCAGCCCCCCGCCTCGATGGACCAACTGCGCCGAAATTCcctaccctacgtcctcctgc
attP_8_M24_F	GAATTTCGGCGCAGTTGGTCCAAGGAGGCGGGGGGCTGGT
attP_8_M24_R	ACCAGCCCCCCGCCTCCTTGGACCAACTGCGCCGAAATTCcctaccctacgtcctcctgc
attP_8_M25_F	GAATTTCGGCGCAGTTGGTCCAACCAGGCGGGGGGCTGGT
attP_8_M25_R	ACCAGCCCCCCGCCTGGTTGGACCAACTGCGCCGAAATTCcctaccctacgtcctcctgc
attP_8_M26_F	GAATTTCGGCGCAGTTGGTCCAACGTGGCGGGGGGCTGGT
attP_8_M26_R	ACCAGCCCCCCGCCACGTTGGACCAACTGCGCCGAAATTCcctaccctacgtcctcctgc
attP_8_M27_F	GAATTTCGGCGCAGTTGGTCCAACGACGCGGGGGGCTGGT
attP_8_M27_R	ACCAGCCCCCCGCGTCGTTGGACCAACTGCGCCGAAATTCcctaccctacgtcctcctgc
attP_8_M28_F	GAATTTCGGCGCAGTTGGTCCAACGAGCCGGGGGGCTGGT
attP_8_M28_R	ACCAGCCCCCCGGCTCGTTGGACCAACTGCGCCGAAATTCcctaccctacgtcctcctgc
attP_8_M29_F	GAATTTCGGCGCAGTTGGTCCAACGAGGGGGGGGGCTGGT
attP_8_M29_R	ACCAGCCCCCCCCCTCGTTGGACCAACTGCGCCGAAATTCcctaccctacgtcctcctgc
attP_8_M30_F	GAATTTCGGCGCAGTTGGTCCAACGAGGCCGGGGGCTGGT
attP_8_M30_R	ACCAGCCCCCGGCCTCGTTGGACCAACTGCGCCGAAATTCcctaccctacgtcctcctgc
attP_8_M31_F	GAATTTCGGCGCAGTTGGTCCAACGAGGCGCGGGGCTGGT
attP_8_M31_R	ACCAGCCCCGCGCCTCGTTGGACCAACTGCGCCGAAATTCcctaccctacgtcctcctgc
attP_8_M32_F	GAATTTCGGCGCAGTTGGTCCAACGAGGCGGCGGGCTGGT
attP_8_M32_R	ACCAGCCCGCCGCCTCGTTGGACCAACTGCGCCGAAATTCcctaccctacgtcctcctgc
attP_8_M33_F	GAATTTCGGCGCAGTTGGTCCAACGAGGCGGGCGGCTGGT
attP_8_M33_R	ACCAGCCGCCCGCCTCGTTGGACCAACTGCGCCGAAATTCcctaccctacgtcctcctgc
attP_8_M34_F	GAATTTCGGCGCAGTTGGTCCAACGAGGCGGGGCGCTGGT
attP_8_M34_R	ACCAGCGCCCCGCCTCGTTGGACCAACTGCGCCGAAATTCcctaccctacgtcctcctgc
attP_8_M35_F	GAATTTCGGCGCAGTTGGTCCAACGAGGCGGGGGCCTGGT
attP_8_M35_R	ACCAGGCCCCCGCCTCGTTGGACCAACTGCGCCGAAATTCcctaccctacgtcctcctgc

attP_8_M36_F	GAATTTCGGCGCAGTTGGTCCAACGAGGCGGGGGGGTGGT
attP_8_M36_R	ACCACCCCCCCGCCTCGTTGGACCAACTGCGCCGAAATTCcctaccctacgtcctcctgc
attP_8_M37_F	GAATTTCGGCGCAGTTGGTCCAACGAGGCGGGGGGCAGGT
attP_8_M37_R	ACCTGCCCCCCGCCTCGTTGGACCAACTGCGCCGAAATTCcctaccctacgtcctcctgc
attP_8_M38_F	GAATTTCGGCGCAGTTGGTCCAACGAGGCGGGGGGCTCGT
attP_8_M38_R	ACGAGCCCCCCGCCTCGTTGGACCAACTGCGCCGAAATTCcctaccctacgtcctcctgc
attP_8_M39_F	GAATTTCGGCGCAGTTGGTCCAACGAGGCGGGGGGCTGCT
attP_8_M39_R	AGCAGCCCCCCGCCTCGTTGGACCAACTGCGCCGAAATTCcctaccctacgtcctcctgc
attP_8_M40_F	GAATTTCGGCGCAGTTGGTCCAACGAGGCGGGGGGCTGGA
attP_8_M40_R	TCCAGCCCCCCGCCTCGTTGGACCAACTGCGCCGAAATTCcctaccctacgtcctcctgc
PrdfS4_1_F	GACTCTTCGCCCGTCCCCAC
PrdfS4_1_R	GTGGGGACGGGCGAAGAGTCcctaccctacgtcctcctgc
PrdfS4_2_F	TTCGCCCGTCCCCACGACGA
PrdfS4_2_R	TCGTCGTGGGGACGGGCGAAcctaccctacgtcctcctgc
PrdfS4_3_F	CCGTCCCCACGACGACCTCG
PrdfS4_3_R	CGAGGTCGTCGTGGGGACGGcctaccctacgtcctcctgc
PrdfS4_4_F	CCCACGACGACCTCGTTCGC
PrdfS4_4_R	GCGAACGAGGTCGTCGTGGGcctaccctacgtcctcctgc
PrdfS4_5_F	GACGACCTCGTTCGCCGATC
PrdfS4_5_R	GATCGGCGAACGAGGTCGTCcctaccctacgtcctcctgc
attP8_1_F	GAATTTCGGCGCAGTTGGTC
attP8_1_R	GACCAACTGCGCCGAAATTCcctaccctacgtcctcctgc
attP8_2_F	TCGGCGCAGTTGGTCCAACG
attP8_2_R	CGTTGGACCAACTGCGCCGAcctaccctacgtcctcctgc
attP8_3_F	GCAGTTGGTCCAACGAGGCG
attP8_3_R	CGCCTCGTTGGACCAACTGCcctaccctacgtcctcctgc
attP8_4_F	TGGTCCAACGAGGCGGGGGG
attP8_4_R	CCCCCCGCCTCGTTGGACCAcctaccctacgtcctcctgc
attP8_5_F	CAACGAGGCGGGGGGCTGGT
attP8_5_R	ACCAGCCCCCCGCCTCGTTGcctaccctacgtcctcctgc

≠The nucleotide changed in the *attP*_8 mutagenesis study is shown in red.
ReDCaT linker shown in lowercase.

Supplemental Table S3: SAXS data collection and analysis statistics for 6H-RdfS-*attP*_8

(*a*) Sample details	
Organism	*Mesorhizobium japonicum R7A*
Source	*E. coli* expression for 6H-RdfS, synthetic oligomer DNA for *attP*_8
Description of complex	6H-RdfS tetramer bound to 40-mer *attP*_8 dsDNA
Scattering particle composition on 6H-RdfS-*attP*_8	
Protein(s)	RdfS (Uniprot Q7AL96) with N-terminus 6H purification tag: MKHHHHHHPMSDYDIPTTENLYFQG
DNA(s)	*attP*_8 40-mer dsDNA: 5'-GAATTTCGGCGCAGTTGGTCCAACGAGGCGGGGGGCTGGT-3'
Stoichiometry of components	4:1 (protein:dsDNA)
Sample environment/configuration on 6H-RdfS-*attP*_8	
Solvent composition	150 mM Tris-HCl, 300 mM NaCl, 5% *v/v* glycerol; pH 7.4
Sample temperature (°C)	25
In beam sample cell	In-line size-exclusion chromatography coupled synchrotron SAXS
Size Exclusion Chromatography (SEC-SAXS) on 6H-RdfS-*attP*_8	
Sample injection concentration, μM	6H-RdfS at 390 μM, *attP*_8 at 500 μM
Sample injection volume, μL	50
SEC column type	Superdex 200 Increase 5/150 GL (GE Healthcare)
SEC flowrate, mL/min	0.3
(*b*) SAS data collection	
Data acquisition/reduction software	Collected and reduced with scatterBrain (Stephen Mudie, Australian Synchrotron)
Source	Pilatus 1M detector
Measured *q*-range ($q_{min} – q_{max}$; $Å^{-1}$)	0.004 – 0.3827
Method for scaling intensities	Linear interpolation of average scattering frames; scattering of the buffer-blank was subtracted during processing
Exposure time(s) & number of exposures	Collected 800 successive 1 second frames (36 frames processed)
Beam geometry (μm)	22
Wavelength (Å)	1.0332
Sample-to-detector distance (m)	2.6
(*c*) SAS-derived structural parameters	
Methods/Software	PRIMUS/qt, AUTORG, GNOM (ATSAS 3.2.1)
Guinier Analysis on 6H-RdfS-*attP*_8	
$I(0) \pm \sigma$ (cm^{-1})	0.086 ± 0.000
$R_g \pm \sigma$ (Å)	38.28 ± 0.07
Guinier range (q^2)	0.0001 – 0.00103
qR_g limits (datapoint range)	0.34 – 1.22 (10-51)
Linear fit assessment (AUTORG fidelity)	1.00
P(r) analysis on 6H-RdfS-*attP*_8	
$I(0) \pm \sigma$ (cm^{-1})	0.086 ± 0.00
$R_g \pm \sigma$ (Å)	39.10 ± 0.09
d_{max} (Å)	141
q-range ($Å^{-1}$)	0.004 – 0.3814
P(*r*) fit assessment (GNOM quality estimate)	0.811

(*d*) Scattering particle size	
Methods/Software	PRIMUS/qt (ATSAS 3.2.1)
Volume estimates on 6H-RdfS-*attP*_8	
Porod volume, V_p (Å^3)	134171
Molecular weight estimates (Da) on 6H-RdfS-*attP*_8	
From chemical composition	76520.58
From SAXS, volume of correlation (V_c)	76135
From SAXS, size and shape	85655
From Bayesian Inference (confidence), credibility interval (Credibility Interval Probability)	83125 (21.55%), 75300-86950 (91.27%)
(*e*) Modelling	
Shape modelling method on 6H-RdfS-*attP*_8	
Software	DAMMIF
q-range for fit ($q_{min} – q_{max}$; Å^{-1})	0.004 – 0.209
Symmetry/anisotropy assumptions	*P*1 (unknown anisometry)
Number of individual model reconstructions	10 models
χ^2, CorMap *P*-values for fit	χ^2 varies per model (0.297 – 0.302)*, *P*-values ~0.0104
Software	DAMAVER
Method	ICP; Iterative Closest Point (point cloud in real space)
Input files	All 10 individual DAMMIF models
ICP mean (standard deviation)	21.37 (± 13.83)
Global inclusion; model clustering	All 10 models included in global analysis; 4 clusters generated
Fourier shell correlation resolution (Å)	42.31
(*f*) Data and model deposition	
SASBDB	SASDW97

*Data collected at the SAXS/WAXS beamline at the Australian Synchrotron and reduced with *scatterBrain* (available at http://archive.synchrotron.org.au/aussyncbeamlines/saxswaxs/software-saxswaxs) has a 2x standard error on the scattered intensity. Therefore, a perfect fit to the experimental data would have a χ^2 value of 0.25 (rather than 1).

Supplemental Table S4. Cluster analysis of unbound simulation trajectories.

**Protein**	**Number of clusters**
Full-length RdfS	26
N-terminally truncated $RdfS_{13\text{-}89}$	183

Supplemental Table S5: ICE*Ml*Sym^{R7A} conjugation frequencies in the presence of truncated RdfS

**Donor**	**Recipient†**	**Conjugation frequency‡**	**Standard deviation**
R7A	R7ANS	1.7 x 10^{-7}	7.5 x 10^{-8}
R7AΔ*qseM*	R7ANS	2.2 x 10^{-4}	2.6 x 10^{-5}
R7AΔ*qseM* pJR202	R7ANS	1.1 x 10^{-4}	1.4 x 10^{-5}
R7AΔ*qseM*Δ*rdfS* pFAJ1700	R7ANS	ND*	-
R7AΔ*qseM*Δ*rdfS* pFAJ1708	R7ANS	ND*	-
R7AΔ*qseM*Δ*rdfS* pJR202	R7ANS	1.2 x 10^{-4}	1.7 x 10^{-5}
R7AΔ*qseM*Δ*rdfS* pFJrdfS$_{13\text{-}89}$	R7ANS	ND*	-
R7AΔ*qseM*Δ*rdfS* pF08rdfS$_{13\text{-}89}$	R7ANS	ND*	-
R7AΔ*qseM*Δ*rdfS* pFJrdfS$_{1\text{-}64}$	R7ANS	1.0 x 10^{-4}	4.4 x 10^{-5}

†All recipients carried pPR3G to enable selection with gentamicin.

‡Mean of three biological replicates expressed as transconjugants per donor.

*Not detected; detection limit ~1.3 x 10^{-9} transconjugants per donor.

Supporting information references

1. Ramsay, J.P., Sullivan, J.T., Stuart, G.S., Lamont, I.L. and Ronson, C.W. (2006) Excision and transfer of the *Mesorhizobium loti* R7A symbiosis island requires an integrase IntS, a novel recombination directionality factor RdfS, and a putative relaxase RlxS. *Mol Microbiol*, **62**, 723-734.
2. Ronson, C.W., Nixon, B.T., Albright, L.M. and Ausubel, F.M. (1987) *Rhizobium meliloti ntrA* (*rpoN*) gene is required for diverse metabolic functions. *J Bacteriol*, **169**, 2424-2431.
3. Beringer, J.E. (1974) R factor transfer in *Rhizobium leguminosarum*. *Microbiology*, **84**, 188-198.
4. Ramsay, J.P., Major, A.S., Komarovsky, V.M., Sullivan, J.T., Dy, R.L., Hynes, M.F., Salmond, G.P.C. and Ronson, C.W. (2013) A widely conserved molecular switch controls quorum sensing and symbiosis island transfer in *Mesorhizobium loti* through expression of a novel antiactivator. *Mol Microbiol*, **87**, 1-13.
5. Ramsay, J.P. (2013) High-throughput β-galactosidase and β-glucuronidase assays using fluorogenic substrates. *Bio Protoc*, **3**, e827.
6. Petoukhov, M.V., Franke, D., Shkumatov, A.V., Tria, G., Kikhney, A.G., Gajda, M., Gorba, C., Mertens, H.D.T., Konarev, P.V. and Svergun, D.I. (2012) New developments in the ATSAS program package for small-angle scattering data analysis. *J Appl Crystallogr*, **45**, 342-350.
7. Konarev, P.V., Volkov, V.V., Sokolova, A.V., Koch, M.H.J. and Svergun, D.I. (2003) PRIMUS: a Windows PC-based system for small-angle scattering data analysis. *J Appl Crystallogr*, **36**, 1277-1282.
8. Semenyuk, A.V. and Svergun, D.I. (1991) GNOM - a program package for small-angle scattering data processing. *J Appl Crystallogr*, **24**, 537-540.
9. Franke, D. and Svergun, D.I. (2009) DAMMIF, a program for rapid *ab-initio* shape determination in small-angle scattering. *J Appl Crystallogr*, **42**, 342-346.
10. Volkov, V.V. and Svergun, D.I. (2003) Uniqueness of *ab initio* shape determination in small-angle scattering. *J Appl Crystallogr*, **36**, 860-864.
11. Franke, D., Petoukhov, M.V., Konarev, P.V., Panjkovich, A., Tuukkanen, A., Mertens, H.D.T., Kikhney, A.G., Hajizadeh, N.R., Franklin, J.M., Jeffries, C.M. *et al.* (2017) ATSAS 2.8: a comprehensive data analysis suite for small-angle scattering from macromolecular solutions. *J Appl Crystallogr*, **50**, 1212-1225.
12. Svergun, D., Barberato, C. and Koch, M.H.J. (1995) CRYSOL - a program to evaluate X-ray solution scattering of biological macromolecules from atomic coordinates. *J Appl Crystallogr*, **28**, 768-773.
13. Hemming, I.A., Clément, O., Gladwyn-Ng, I.E., Cullen, H.D., Ng, H.L., See, H.B., Ngo, L., Ulgiati, D., Pfleger, K.D.G., Agostino, M. *et al.* (2019) Disease-associated missense variants in ZBTB18 disrupt DNA binding and impair the development of neurons within the embryonic cerebral cortex. *Hum Mutat*, **40**, 1841-1855.
14. Schumacher, M.A., den Hengst, C.D., Bush, M.J., Le, T.B.K., Tran, N.T., Chandra, G., Zeng, W., Travis, B., Brennan, R.G. and Buttner, M.J. (2018) The MerR-like protein BldC binds DNA direct repeats as cooperative multimers to regulate *Streptomyces* development. *Nat Commun*, **9**, 1139.
15. Rice, P., Longden, I. and Bleasby, A. (2000) EMBOSS: the European Molecular Biology Open Software Suite. *Trends Genet*, **16**, 276-277.
16. Salomon-Ferrer, R., Case, D.A. and Walker, R.C. (2013) An overview of the Amber biomolecular simulation package. *WIREs Comput Mol Sci*, **3**, 198-210.
17. Kohnke, B., Kutzner, C. and Grubmüller, H. (2020) A GPU-accelerated fast multipole method for GROMACS: performance and accuracy. *J Chem Theory Comput*, **16**, 6938-6949.

18. Tribello, G.A., Bonomi, M., Branduardi, D., Camilloni, C. and Bussi, G. (2014) PLUMED 2: New feathers for an old bird. *Comput Phys Commun*, **185**, 604-613.
19. Maier, J.A., Martinez, C., Kasavajhala, K., Wickstrom, L., Hauser, K.E. and Simmerling, C. (2015) ff14SB: Improving the Accuracy of Protein Side Chain and Backbone Parameters from ff99SB. *J Chem Theory Comput*, **11**, 3696-3713.
20. Pérez, A., Marchán, I., Svozil, D., Sponer, J., Cheatham, T.E., Laughton, C.A. and Orozco, M. (2007) Refinement of the amber force field for nucleic acids: improving the description of α/γ conformers. *Biophys J*, **92**, 3817-3829.
21. Zgarbová, M., Luque, F.J., Sponer, J., Cheatham, T.E., 3rd, Otyepka, M. and Jurečka, P. (2013) Toward improved description of DNA backbone: revisiting epsilon and zeta torsion force field parameters. *J Chem Theory Comput*, **9**, 2339-2354.
22. Krepl, M., Zgarbová, M., Stadlbauer, P., Otyepka, M., Banáš, P., Koča, J., Cheatham, T.E., 3rd, Jurečka, P. and Sponer, J. (2012) Reference simulations of noncanonical nucleic acids with different χ variants of the AMBER force field: quadruplex DNA, quadruplex RNA and Z-DNA. *J Chem Theory Comput*, **8**, 2506-2520.
23. Sousa da Silva, A.W. and Vranken, W.F. (2012) ACPYPE - AnteChamber PYthon Parser interfacE. *BMC Red Notes*, **5**, 367.
24. Jorgensen, W.L., Chandrasekhar, J., Madura, J.D., Impey, R.W. and Klein, M.L. (1983) Comparison of simple potential functions for simulating liquid water. *J Chem Phys*, **79**, 926-935.
25. Joung, I.S. and Cheatham, T.E., III. (2008) Determination of alkali and halide monovalent ion parameters for use in explicitly solvated biomolecular simulations. *J Phys Chem B*, **112**, 9020-9041.
26. Bussi, G., Donadio, D. and Parrinello, M. (2007) Canonical sampling through velocity rescaling. *J Chem Phys*, **126**, 014101.
27. Berendsen, H.J.C., Postma, J.P.M., van Gunsteren, W.F., DiNola, A. and Haak, J.R. (1984) Molecular dynamics with coupling to an external bath. *J Chem Phys*, **81**, 3684-3690.
28. Essmann, U., Perera, L., Berkowitz, M.L., Darden, T., Lee, H. and Pedersen, L.G. (1995) A smooth particle mesh Ewald method. *J Chem Phys*, **103**, 8577-8593.
29. Hess, B., Bekker, H., Berendsen, H.J.C. and Fraaije, J.G.E.M. (1997) LINCS: A linear constraint solver for molecular simulations. *J Comput Chem*, **18**, 1463-1472.
30. Marchi, M. and Ballone, P. (1999) Adiabatic bias molecular dynamics: A method to navigate the conformational space of complex molecular systems. *J Chem Phys*, **110**, 3697-3702.
31. Daura, X., Gademann, K., Jaun, B., Seebach, D., van Gunsteren, W.F. and Mark, A.E. (1999) Peptide folding: when simulation meets experiment. *Angew Chem Int Ed*, **38**, 236-240.
32. Newberry, K.J. and Brennan, R.G. (2004) The structural mechanism for transcription activation by MerR family member multidrug transporter activation, N terminus. *J Biol Chem*, **279**, 20356-20362.
33. Abbani, M., Iwahara, M. and Clubb, R.T. (2005) The Structure of the Excisionase (Xis) Protein from Conjugative Transposon Tn*916* Provides Insights into the Regulation of Heterobivalent Tyrosine Recombinases. *J Mol Biol*, **347**, 11-25.
34. Sam, M.D., Cascio, D., Johnson, R.C. and Clubb, R.T. (2004) Crystal structure of the excisionase–DNA complex from bacteriophage lambda. *J Mol Biol*, **338**, 229-240.
35. Berntsson, R.P., Odegrip, R., Sehlén, W., Skaar, K., Svensson, L.M., Massad, T., Högbom, M., Haggård-Ljungquist, E. and Stenmark, P. (2014) Structural insight into DNA binding and oligomerization of the multifunctional Cox protein of bacteriophage P2. *Nucleic Acids Res*, **42**, 2725-2735.
36. Colombi, E., Perry, B.J., Sullivan, J.T., Bekuma, A.A., Terpolilli, J.J., Ronson, C.W. and Ramsay, J.P. (2021) Comparative analysis of integrative and conjugative mobile genetic elements in the genus *Mesorhizobium*. *Microb Genom*, **7**.

37. Madeira, F., Madhusoodanan, N., Lee, J., Eusebi, A., Niewielska, A., Tivey, A.R.N., Lopez, R. and Butcher, S. (2024) The EMBL-EBI Job Dispatcher sequence analysis tools framework in 2024. *Nucleic Acids Res*, **52**, W521-W525.
38. Bond, C.S. and Schüttelkopf, A.W. (2009) ALINE: a WYSIWYG protein-sequence alignment editor for publication-quality alignments. *Acta Crystallogr D Biol Crystallogr*, **65**, 510-512.
39. Thoma, S. and Schobert, M. (2009) An improved *Escherichia coli* donor strain for diparental mating. *FEMS Microbiol Lett*, **294**, 127-132.
40. Studier, F.W. and Moffatt, B.A. (1986) Use of bacteriophage T7 RNA polymerase to direct selective high-level expression of cloned genes. *J Mol Biol*, **189**, 113-130.
41. Robichon, C., Luo, J., Causey, T.B., Benner, J.S. and Samuelson, J.C. (2011) Engineering *Escherichia coli* BL21(DE3) derivative strains to minimize *E. coli* protein contamination after purification by immobilized metal affinity chromatography. *Appl Environ Microbiol*, **77**, 4634-4646.
42. Sullivan, J., Patrick, H., Lowther, W., Scott, D. and Ronson, C. (1995) Nodulating strains of *Rhizobium loti* arise through chromosomal symbiotic gene transfer in the environment. *Proc Natl Acad Sci USA*, **92**, 8985.
43. Verdonk, C.J., Sullivan, J.T., Williman, K.M., Nicholson, L., Bastholm, T.R., Hynes, M.F., Ronson, C.W., Bond, C.S. and Ramsay, J.P. (2019) Delineation of the integrase-attachment and origin-of-transfer regions of the symbiosis island ICE*Ml*Sym^{R7A}. *Plasmid*, **104**, 102416.
44. Dümmler, A., Lawrence, A.M. and de Marco, A. (2005) Simplified screening for the detection of soluble fusion constructs expressed in *E. coli* using a modular set of vectors. *Microb Cell Fact*, **4**.
45. Verdonk, C.J., Marshall, A.C., Ramsay, J.P. and Bond, C.S. (2022) Crystallographic and X-ray scattering study of RdfS, a recombination directionality factor from an integrative and conjugative element. *Acta Crystallogr D*, **78**, 1210-1220.
46. Khan, S.R., Gaines, J., Roop, R.M., 2nd and Farrand, S.K. (2008) Broad-host-range expression vectors with tightly regulated promoters and their use to examine the influence of TraR and TraM expression on Ti plasmid quorum sensing. *Appl Environ Microbiol*, **74**, 5053-5062.
47. Dombrecht, B., Vanderleyden, J. and Michiels, J. (2001) Stable RK2-derived cloning vectors for the analysis of gene expression and gene function in gram-negative bacteria. *Mol Plant Microbe Interact*, **14**, 426-430.
48. Ramsay, J.P., Tester, L.G.L., Major, A.S., Sullivan, J.T., Edgar, C.D., Kleffmann, T., Patterson-House, J.R., Hall, D.A., Tate, W.P., Hynes, M.F. *et al.* (2015) Ribosomal frameshifting and dual-target antiactivation restrict quorum-sensing-activated transfer of a mobile genetic element. *Proc Natl Acad Sci USA*, **112**, 4104.
49. Antoine, R., Alonso, S., Raze, D., Coutte, L., Lesjean, S., Willery, E., Locht, C. and Jacob-Dubuisson, F. (2000) New virulence-activated and virulence-repressed genes identified by systematic gene inactivation and generation of transcriptional fusions in *Bordetella pertussis*. *J Bacteriol*, **182**, 5902-5905.
50. Rodpothong, P., Sullivan, J.T., Songsrirote, K., Sumpton, D., Cheung, K.W., Thomas-Oates, J., Radutoiu, S., Stougaard, J. and Ronson, C.W. (2009) Nodulation gene mutants of *Mesorhizobium loti* R7A-*nodZ* and *nolL* mutants have host-specific phenotypes on *Lotus* spp. *Mol Plant Microbe Interact*, **22**, 1546-1554.
51. Miller, W.G., Leveau, J.H. and Lindow, S.E. (2000) Improved *gfp* and *inaZ* broad-host-range promoter-probe vectors. *Mol Plant Microbe Interact*, **13**, 1243-1250.
52. Ramsay, J.P., Bastholm, T.R., Verdonk, C.J., Tambalo, D.D., Sullivan, John T., Harold, Liam K., Panganiban, B.A., Colombi, E., Perry, Benjamin J., Jowsey, W. *et al.* (2021) An epigenetic switch activates bacterial quorum sensing and horizontal transfer of an integrative and conjugative element. *Nucleic Acids Res*, **50**, 975-988.
